# The trihelix family of transcription factors: functional and evolutionary analysis in Moso bamboo (*Phyllostachys edulis*)

**DOI:** 10.1186/s12870-019-1744-8

**Published:** 2019-04-25

**Authors:** Xinran Cheng, Rui Xiong, Hanwei Yan, Yameng Gao, Huanlong Liu, Min Wu, Yan Xiang

**Affiliations:** 1 Laboratory of Modern Biotechnology, School of Forestry and Landscape Architecture, Anhui Agricultural University, Hefei, 230036 China; 2 National Engineering Laboratory of Crop Stress Resistance Breeding, Anhui Agricultural University, Hefei, 230036 China; 3 Key Laboratory of Crop Biology of Anhui Province, School of Forestry and Landscape Architecture, Anhui Agricultural University, Hefei, 230036 China

**Keywords:** Trihelix transcription factors, Gene ontology, Phylogenetic analysis, Expression profiling, Moso bamboo, Subcellular localization, Transcriptional activity assay

## Abstract

**Background:**

Trihelix transcription factors (*TTFs*) are photoresponsive proteins that have a representative three-helix structure (helix-loop-helix-loop-helix). Members of this gene family have been reported to play roles in many plant processes.

**Results:**

In this study, we performed a functional and evolutionary analysis of the TTFs in Moso bamboo (*Phyllostachys edulis)*. A total of 35 genes were identified and grouped into five subfamilies (*GT-1, GT-γ, GT-2, SIP1* and *SH4*) according to their structural properties. Gene structure analysis showed that most genes in the *PeTTF* family had fewer introns. A unique motif (Motif 16) to the *GT-γ* subfamily was identified by conserved motif analysis. Promoter analysis revealed various cis-acting elements related to plant growth and development, abiotic and biotic stresses, and phytohormone responses. Data for the 35 Moso bamboo *TTF* genes were used to generate heat maps, which indicated that these genes were expressed in different tissues or developmental stages. Most of the *TTF* genes identified here had high expression in leaves and panicles according to the expression profile analysis. The expression levels of the *TTF* members in young leaves were studied using quantitative real-time PCR to determine their tissue specificity and stress-related expression patterns to help functionally characterize individual members.

**Conclusions:**

The results indicated that members of the *TTF* gene family may be involved in plant responses to stress conditions. Additionally, PeTTF29 was shown to be located in the nucleus by subcellular localization analysis and to have transcriptional activity in a transcriptional activity assay. Our research provides a comprehensive summary of the *PeTTF* gene family, including functional and evolutionary perspectives, and provides a basis for functionally characterizing these genes.

**Electronic supplementary material:**

The online version of this article (10.1186/s12870-019-1744-8) contains supplementary material, which is available to authorized users.

## Background

Transcription factors are a class of DNA-binding proteins that interact specifically with cis-acting elements in the promoter regions of target genes; they generally contain DNA-binding domains, transcriptional activation domains, and nuclear localization signals and can either inhibit or induce the transcription of target genes. [1]. There are more than 60 transcription factor gene families in plants [2]. Recently, many researchers have focused on the trihelix transcription factor (*TTF*) gene family because its members contain a unique DNA-binding domain including three helical structures (helix-loop-helix-loop-helix). This family is also referred to as the *GT* transcription factor family, whose core sequence is 5′-G-Pu-(T/A)-A-A-(T/A)-3′ and members of which bind to the photo-responsive element *GT* in DNA. [3–7].

Since the beginning of this century, whole genome sequencing has been completed for many plants and the important functions of a series of gene families have been determined. Research on the *TTF* gene family has been gradually carried out, functional investigation of the family has widened, and it has become the subject of much recent attention from biologists. [2, 4, 8–11]. To data, 20, 36, 31, 28, 56, 63 and 59 *TTF* genes have been identified in chrysanthemum (*Chrysanthemum morifolium*), tomato (*Lycopersicon esculentum*), rice (*Oryza sativa*), Arabidopsis (*Arabidopsis thaliana*), poplar (*Populus*), soybean (*Glycine max*) and maize (*Zea mays*), respectively [3–7, 12, 13]. The earliest identified *TTF* gene was the pea *GT-1* protein factor, which binds specifically to the GT element in the pea nucleus. Later, Lam et al. cloned the tobacco *GT-1* factor and confirmed that it contained three α-helix domains in series [4, 8]. Nevertheless, the *TTF* gene family in Moso bamboo has not been comprehensively studied.

There is growing evidence that *TTF* genes play crucial roles in plant growth and development [14], For instance, the development of petals and sepals, and sepal fusion are regulated by the Arabidopsis PETAL LOSS (PTL) genes (GT-2 subfamily) [15]. A previous study on poplar reported that *PtaGTL1* was highly homologous to Arabidopsis *AtGTL1* and was involved the development of stomata and trichomes [16]. The expression of *SHAT1* in the shedding zone is regulated by the triangular leaf transcription factor *SH4*, which is involved in regulating grain separation traits in rice [17]. Additionally, Arabidopsis *At3g10030* was associated with leaf development because the corresponding mutants exhibited leaves that were small, deformed and light green [16]. The key roles *TTF* genes play in responses to biotic and abiotic stresses have also been well documented [4, 14, 18, 19]. Fang et al. reported that the rice gene *GTγ-1* enhanced resistance to salt stress and was slightly induced by ABA treatment and drought stress; in addition, *GTγ-2* and *GTγ-3* were strongly induced by most abiotic stresses [4]. Furthermore, overexpression in transgenic Arabidopsis plants demonstrated that *GmGT-2A* and *GmGT-2B* could enhance tolerance to salt, drought and freezing stresses [20]. On the whole, trihelix transcription factors play important roles in the regulation of developmental processes.

Moso bamboo is a rare species of evergreen tree-like bamboo that grows rapidly [21], and has gradually become widely used and an economically important Gramineae species in China [22]. However, Moso bamboo trees are affected by a variety of environmental stresses, including the rapid spread of pests and drought stress, which lead to significant economic losses. Increasing the resistance of Moso bamboo to these stresses to improve both the quality and quantity of bamboo produced is one of the major aims of researchers working in this area [23, 24].

In this study, we identified 35 members of the *PeTTF* gene family and conducted various bioinformatics analyses, including phylogenetic, gene structure, expression profile, conserved motif, gene ontology annotation, subcellular localization and promoter cis-acting regulatory element analyses. Moreover, the 35 *PeTTF* genes were analyzed by qRT-PCR to study their responses to different stresses, including MeJA treatment, and drought and salt stress. We found that all of the genes were stress responsive. The objective of this study was to determine the transcriptional responses of *TTF* gene family members to various stresses and plant hormone treatments.

## Materials and methods

### Database searches and identification of *TTF* family genes in Moso bamboo

The accession numbers of Arabidopsis trihelix family members were acquired from PlantTFDB (http://planttfdb.cbi.pku.edu.cn) [25], which is a comprehensive database that provides a complete list of transcription factors in plant species. Rice trihelix protein sequences were obtained from the rice genome annotation database (http://rice.plantbiology.msu.edu/analyses_search_locus.shtml) [26].

The genome sequence of Moso bamboo was downloaded through the Bamboo Genome Database (http://www.bamboogdb.org/). A hidden Markov model (HMM) profile based on the *TTF* (PF13837) domain was used to perform a local BLAST search (E-value-5) of the Pfam database (http://pfam.janelia.org/search/sequence) [18, 27, 28]. All of the selected Moso bamboo sequences were filtered, leaving only candidate genes containing known conserved domains, which were checked using the Pfam database (http://pfam.janelia.org/), the NCBI Conserved Domain database (http://www.ncbi.nlm.nih.gov/Structure/cdd/wrpsb.cgi), and the SMART database (http://smart.embl-heidelberg.de/). ExPASy (http://www.expasy.ch/tools/pi_tool.html) was used to determine the amino acid sequence, open reading frame (ORF) length, molecular weight (MW) and isoelectric point (pI) [29] and perform bioinformatics analysis of each Moso bamboo *TTF* gene. Additionally, gene identifiers and genomic/coding/protein sequences were acquired from the Bamboo Genome Database (http://www.bamboogdb.org/).

### Phylogenetic analysis

Further multiple sequence alignments of all TTF proteins of Moso bamboo were performed using ClustalX 2.11. At the same time, sequence alignments between Moso bamboo, rice and Arabidopsis were also carried out [30, 31]. Phylogenetic trees were constructed in MEGA6.0 [32] using the neighbor-joining method with 1000 repeats used for bootstrap analysis. We obtained the protein sequence of PeTTF gene in the Bamboo Genome Database (http://www.bamboogdb.org/), and then we used the sequence information to construct phylogenetic trees.

### Gene structure and conserved motif analysis

The exon/intron organization of individual *TTF* genes was determined using the GSDS program (http://gsds.cbi.pku.edu.cn/) [33] to compare each cDNA to its corresponding genomic DNA sequence. Motifs shared between related proteins within the *PeTTF* family were identified using the MEME motif search tool (http://meme-suite.org/tools/meme) [34], with the maximum number of motifs identified set at 20 and the maximum width at 300 aa. The sequences of the *TTF* subfamily members were examined using the DNAMAN software and manually modified, with the conserved motifs annotated according to the MEME analysis.

### Identification of homologous and calculation of Ka/Ks values

Paralogous and orthologous genes were identified according to Blanc and Wolfe [35]. For each species, full-pairing nucleotide sequence similarity searches were performed with the transcribed sequences using BlastN [36]. Sequences that had over 300 bp aligned and showed ≥40% identity were defined as paralogous. To identify putative orthologs between two species (A and B), each sequence of species A was searched against all the sequences of species B using BlastN. Then, each sequence from species B was searched against all sequences from species A. If two sequences were the best hit to each other and if more than 300 bp of the two sequences was aligned, the two sequences were defined as orthologous.

Multiple sequence alignment of the homologous (orthologous and paralogous) *TTF* gene pairs was performed using the ClustalX software (version 2.11) software. The sequences were then further aligned using MEGA6.0 and the DnaSP 5 software [37, 38] was used to calculate the Ks and Ka substitution rates. The Ks rate was considered to be an indicator of the time of gene-pair duplication events, and the divergence times (T) were calculated using the Ks value of the λ permutation for each synonymous site per year according to the formula T = Ks/2λ (λ = 6.5 × 10^− 9^) [21, 39, 40]. Then, the Ka/Ks ratios for all homologous gene pairs were determined using a sliding window analysis with the window size set at 150 bp and the step size at 9 bp.

### Expression profile analysis

Expression profile data for each gene was downloaded from the NCBI short read (SRA) database (https://trace.ncbi.nlm.nih.gov/Traces/sra/?study=ERP001341), and then the original RNA-seq reads were trimmed using BioProject ERP001341 to eliminate low quality base calls (Q < 20) and adapter sequences with the pipeline Fastq clean [41]. The differentially expressed genes were then examined by the pipeline tophat2 with default parameters to map the matched clean reads to the *P. bridla* reference genome [42]. Finally, the Heatmapper Plus tool was used to produce heatmaps from the whole-genome microarray data for three biological replicates of seven different tissue specimens including L (leaf), P1 (early panicle), P2 (advanced panicle), R (root), RH (rhizome), S1 (20 cm shoots) and S2 (50 cm shoots) at various developmental phases [43].

### Promoter cis-acting regulatory element analysis, gene ontology (GO) annotation and subcellular localization prediction

The 2000 bp sequence upstream/downstream of each predicted gene coding region was obtained from the Bamboo Genome Database (http:// www.bamboogdb.org/) [44] and analyzed using PlantCARE (http://bioinformatics.psb.ugent.be/webtools/plantcare/html/) [45]. The PlantCARE database is a tool for analyzing plant cis-acting regulatory elements and promoter sequences to identify specific motifs for plant growth and development, phytohormone responses and abiotic and biotic stress responses.

Functional annotation of the *TTF* genes was performed using the Gene Ontology database (GO; www.geneontology.org) [46].

The subcellular localization of the proteins was predicted using WOLF PSORT (http://www.genscript.com/psort.html) [47].

### Plant materials, growth conditions, and stress treatments

The seeds of Moso bamboo from Tianmu Mountain National Nature Reserve in Zhejiang Province were planted in black soil and vermiculite. During their development and growth, the plants were grown in a greenhouse at 25/27 °C and 80% humidity with 16/8 h of light/dark. The plants were manure once every 3 days and watered once a day and cultured for 3 months.

For drought and salt stress treatments, the plants were treated with a 20% PEG-6000 solution [48] and 200 mM NaCl, respectively [49]. The effects of injury were simulated at 25 °C using 100 μM MeJA (Sigma, St. Louis, MO, USA) solution [12]. The plants we treated with stress were annual Moso bamboo. The specific process was as follows: i, the required treatment solution was configured; ii, the configured solution was poured into the pot; iii, 6 periods were collected (0, 1, 3, 6, 12 and 24 h) of Moso bamboo leaves, with 0 h being the control; iv, leaf tissue was immediately frozen in liquid nitrogen and stored at − 80 °C for RNA extraction.

### RNA isolation and quantitative real-time PCR (qRT-PCR) analysis

To confirm the expression levels of the *TTF* genes in Moso bamboo, qRT-PCR analysis using SYBR-green fluorescence was performed for each member. Total RNA was extracted from the leaf, young leaf samples using Trizol reagent (Sangon Biotech, Shanghai, China) according to the manufacturer’s instructions; and an optimized modified cetyl trimethyl ammonium bromide procedure [24] was used to extracted total RNA from Stem, Shoot, root, and Rhizome; gel electrophoresis (1% agar) was used to examine the integrity of the RNA. Then, First-strand cDNA was synthesized using a PrimeScriptTM RT Reagent Kit (TaKaRa). Gene-specific primers were designed using Primer Express 3.0 and the NCBI primer Blast tool and the internal control was performed using the tonoplast intrinsic protein 41 (TIP41) [50].

qRT-PCR was carried out in 20 μl volumes consisting of 10 μl 2× SYBR® PremixExTaq™ (TaKaRa), 0.4 μl 50× ROX Reference Dye, 2 μl diluted cDNA template, 0.8 μl of each specific primer, and 6 μl ddH_2_O. The cycling parameters were 95 °C for 30 s followed by 40 cycles of 95 °C denaturation for 5 s and annealing at 55–60 °C annealing for 34 s. Three biological and three technical replicates were included for each sample. The 2^−ΔΔCT^ [51] method was used to calculate the relative expression level of each gene with expression normalized to the 0 h time point, which was set at 1 [52]. Statistical analyses were conducted using the GraphPad software [53].

### Subcellular localization assay

The full-length CDS of *PeTTF29* was cloned from bamboo, and then the *PeTTF29* coding region was amplified by PCR using the primers 5′-(tgc-*ACTAGT*-ATGGAGGGGAATTTGC)-3′ and 5′-(cgc-*CCCGGG*-CTTCTTTGGTTTGACA)-3′ to introduce *Spe*I and *Sma*I sites, respectively. The product was then cloned into the *pCambia1305* vector (Clontech, Beijing, China) containing the *CaMV 35S* promoter and the *GFP* gene to produce a *PeTTF29-GFP* construct. The constructed *PeTTF29-GFP* vector was inserted into *Agrobacterium tumefaciens* EHA105 by freeze-thawing. The suspension was infiltrated into the *Nicotiana tabacum* leaves using an injection method and the green fluorescent protein (*GFP*) fluorescence was observed using confocal microscopy [54]. *pCambia1305* with only constitutive GFP was used as a control vector.

### Transcription activation assay

The full-length CDS of PeTTF29 was cloned from bamboo, and then the PeTTF29 coding region was amplified by PCR using the primers 5′-(tgc-GAATTC-ATGGAGGGGAATTTGC)-3′ and 5′-(cgc-CTGCAG-AATCTTCTTTGGTTTG)-3′ to introduce EcoRI and PstI sites, respectively. The constructed vector plasmids (target gene, negative control and positive control vector) were transformed into prepared yeast competent cells, and plated on SD/Trp − (as control) and SD/Trp−/His−/Ade−/X-α-gal media. Each plate was neatly divided into three regions containing the target gene, negative control and positive control. The autologous activity of the PeTTF29 gene was verified by streaking.

## Results

### Identification of *TTF* genes in Moso bamboo

We identified 35 members of the *PeTTF* gene family, designated *PeTTF1* to *PeTTF35*. Information about the Moso bamboo *TTF* genes, including their gene identifiers, locations, gene lengths, and CDS lengths, is shown in Table 1. The CDSs ranged from 477 bp (*PeTTF35*) to 1935 (*PeTTF24*) bp in length, with an average length of 1097 bp, and the predicted protein lengths ranged from 158 (*PeTTF35*) to 644 (*PeTTF24*) aa in length, with an average length of 364 aa. The predicted molecular weights ranged from 6250.04 Da (*PeTTF17*) to 70,113.15 Da (*PeTTF31*), with a mean value of 39,553.91 Da. The pI values of all *PeTTF* gene products were below 11, with most falling between 5.0 and 10.0. The pI values of two predicted proteins (*PeTTF33* and *PeTTF35*) were below 5.0, and only two proteins had a pI above 10.0 (*PeTTF2* and *PeTTF8*).

**Table 1:** Detailed information about the predicted *PeTTF* genes

Name	Gene ID	Location	ORF length (bp)	Predicted Protein			Exons
				Size (aa)	MW (Da)	pI	
PeTTF1	PH01000001G0500	PH01000001:348404–353,246	1392	463	51,339.99	6.51	6
PeTTF2	PH01000010G0450	PH01000010:333451–334,674	837	278	31,526.86	10.51	2
PeTTF3	PH01000016G1050	PH01000016:832203–835,604	978	325	35,416.69	6.71	4
PeTTF4	PH01000017G1770	PH01000017:1223108–1,227,861	1632	543	57,598.78	6.66	4
PeTTF5	PH01000019G0430	PH01000019:338352–344,501	1776	591	64,500.43	8.28	7
PeTTF6	PH01000019G0630	PH01000019:498070–502,996	1251	416	44,129.17	6.53	6
PeTTF7	PH01000020G1490	PH01000020:1016528–1,021,991	1161	386	42,551.58	6.42	5
PeTTF8	PH01000023G2170	PH01000023:1389503–1,390,380	591	196	21,556.89	10.89	1
PeTTF9	PH01000042G1290	PH01000042:820202–822,366	1632	543	59,326.99	5.33	1
PeTTF10	PH01000110G0890	PH01000110:616131–618,576	522	173	18,904.82	10.00	1
PeTTF11	PH01000225G1300	PH01000225:784587–789,545	813	270	31,899.86	8.69	2
PeTTF12	PH01000265G1050	PH01000265:729361–733,771	1266	421	45,424.90	9.18	8
PeTTF13	PH01000551G0750	PH01000551:454513–457,660	1317	438	49,565.35	6.18	1
PeTTF14	PH01000749G0800	PH01000749:497748–502,389	816	271	29,897.93	9.04	6
PeTTF15	PH01000778G0550	PH01000778:319370–322,783	942	313	34,196.71	9.96	1
PeTTF16	PH01000823G0680	PH01000823:401827–404,100	1140	379	43,468.32	9.03	1
PeTTF17	PH01000907G0490	PH01000907:299223–301,005	690	229	6250.04	9.30	2
PeTTF18	PH01001050G0190	PH01001050:145304–146,705	609	202	22,559.24	9.84	2
PeTTF19	PH01001160G0450	PH01001160:304848–307,615	732	243	27,274.24	6.72	3
PeTTF20	PH01001191G0080	PH01001191:45575–48,488	1026	341	34,296.75	6.88	1
PeTTF21	PH01001215G0530	PH01001215:388790–394,765	1695	564	62,006.92	9.62	6
PeTTF22	PH01001227G0510	PH01001227:318856–320,192	981	326	35,750.42	6.81	1
PeTTF23	PH01001447G0230	PH01001447:145073–149,866	1059	352	37,733.79	5.19	3
PeTTF24	PH01001451G0080	PH01001451:28239–32,258	1935	644	70,109.82	5.76	3
PeTTF25	PH01001538G0330	PH01001538:231626–234,572	1047	348	38,256.31	6.22	3
PeTTF26	PH01001567G0130	PH01001567:89093–92,345	756	251	27,067.65	9.81	1
PeTTF27	PH01001778G0330	PH01001778:262830–265,479	1287	428	48,983.77	6.27	1
PeTTF28	PH01002213G0310	PH01002213:194429–199,841	1485	494	54,202.70	8.38	7
PeTTF29	PH01002648G0060	PH01002648:37644–39,402	1200	399	46,125.49	6.13	1
PeTTF30	PH01003510G0200	PH01003510:123649–126,664	1329	442	50,199.08	6.35	1
PeTTF31	PH01008287G0010	PH01008287:1698–5966	1926	641	70,113.15	5.60	3
PeTTF32	PH01011203G0010	PH01011203:229–1679	600	199	22,030.79	6.53	3
PeTTF33	PH01047850G0010	PH01047850:55–1624	897	298	31,134.39	4.85	3
PeTTF34	PH01107020G0010	PH01107020:53–1034	624	207	21,063.61	9.94	3
PeTTF35	PH01153193G0010	PH01153193:1–820	477	158	17,923.39	4.68	2

### Phylogenetic analysis of the *TTF* genes in Moso bamboo

**Fig. 1:**
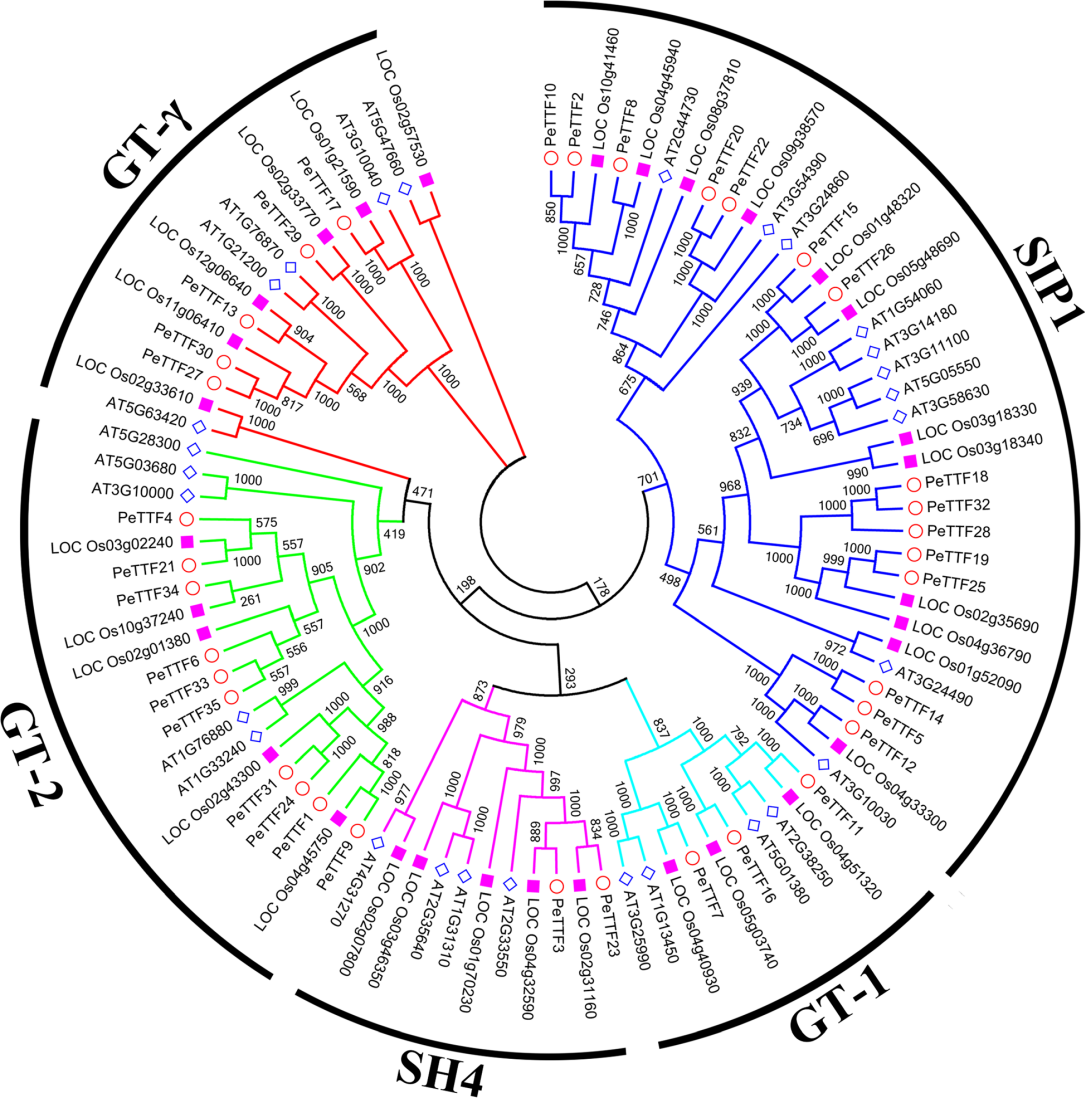
Phylogeny of TTFs from Moso bamboo, rice and Arabidopsis. The 35 PeTTF genes, 31 OsTTF genes and 28 AtTTF genes are clustered into five subfamilies. Details of the TTF genes from Arabidopsis and rice are listed in Additional file 1: Table S1. The tree was generated with the Clustal X 2.0 software using the neighbor-joining (N-J) method

**Fig. 2:**
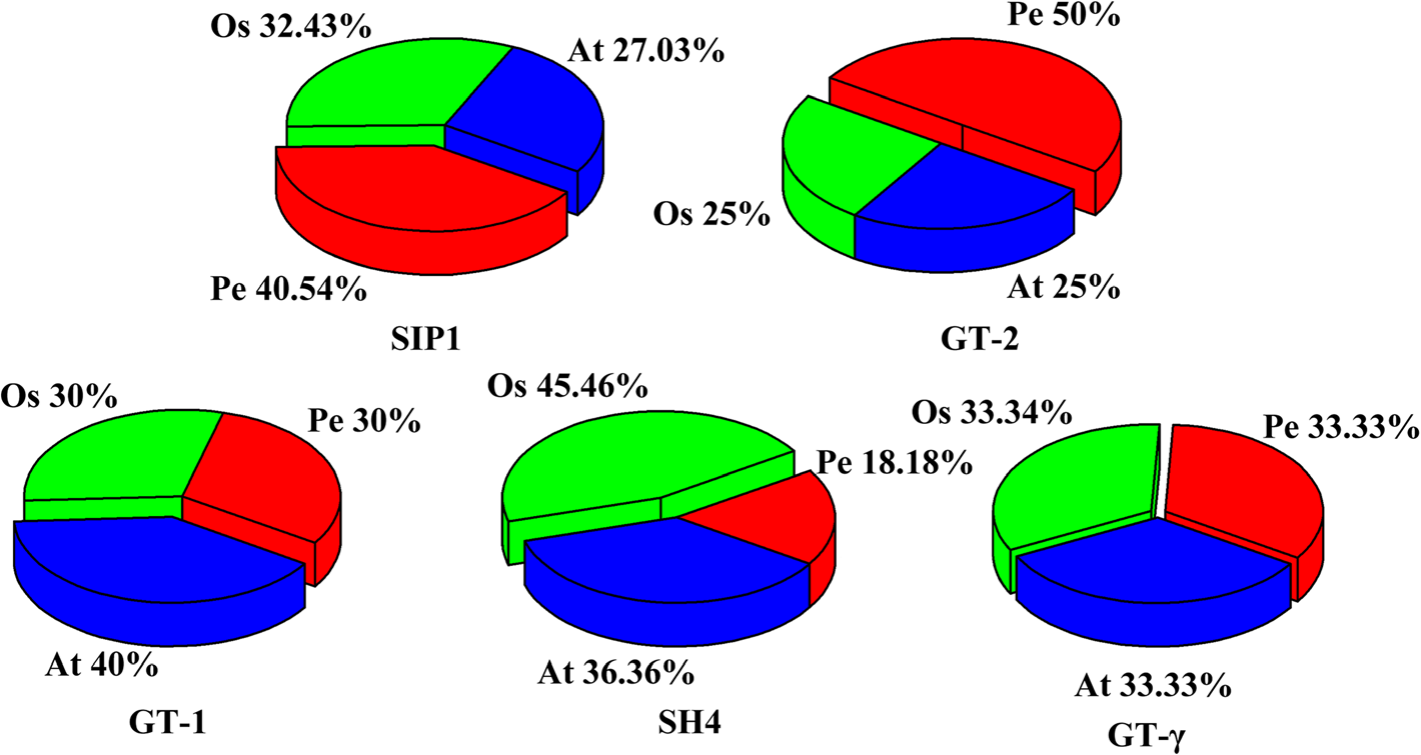
Comparison of TTF family members from Moso bamboo, rice and Arabidopsis. Different colors represent the different species, and the percentage of each subfamily in each species is shown

To explore the phylogenetic relationships among the TTF proteins in rice, Arabidopsis and Moso bamboo, we constructed a neighbor-joining phylogenetic tree with ClustalX using 94 *TTF* sequences, including 31, 28 and 35 sequences from rice, Arabidopsis and Moso bamboo, respectively. The characteristics of these genes are listed in Additional file 1: Table S1. The phylogenetic tree clearly divided the 94 *TTF* genes into five distinct subfamilies according to the bootstrap support and evolutionary distances (Fig. 1). The *SIP1* subfamily contained the largest number of genes, followed by *GT-2, GT-γ* and *SH4*; *GT-1* had the fewest *TTF* genes. The percentage of each subfamily derived from each of the three plant species was calculated (Fig. 2) The *GT-γ* subfamily was evenly distributed among the three species while two of the larger subfamilies (*SIP1* and *GT-2*) contained mostly Moso bamboo genes. However, the *GT-γ* and *SH4* subfamilies were highly represented by Arabidopsis and rice genes, respectively.

We identified paralogous and orthologous genes from the three species, which are listed in Table 2. Fifteen pairs of orthologous genes were identified in rice and Moso bamboo, but there were no orthologs between Moso bamboo and Arabidopsis. Thus, we concluded that the *TTF* genes in the two monocots (Moso bamboo and rice) had closer relationships than with those of the dicotyledons (Arabidopsis), which was consistent with the evolutionary relationship between dicots and monocots.

**Table 2:** Paralogous (Pe-Pe) and orthologous (Pe-Os and Pt-At) gene pairs

Pe-Pe	Pe-Os
PeTTF2/PeTTF10	PeTTF3/LOC_Os04g32590
PeTTF3/PeTTF23	PeTTF7/LOC_Os04g40930
PeTTF4/PeTTF21	PeTTF8/LOC_Os04g45940
PeTTF5/PeTTF14	PeTTF9/LOC_Os04g45750
PeTTF7/PeTTF16	PeTTF11/LOC_Os04g51320
PeTTF15/PeTTF26	PeTTF12/LOC_Os04g33300
PeTTF18/PeTTF32	PeTTF13/LOC_Os12g06640
PeTTF19/PeTTF25	PeTTF15/LOC_Os05g48320
PeTTF20/PeTTF22	PeTTF16/LOC_Os05g03740
PeTTF24/PeTTF31	PeTTF17/LOC_Os01g21590
PeTTF27/PeTTF30	PeTTF21/LOC_Os03g02240
PeTTF33/PeTTF35	PeTTF23/LOC_Os02g31160
	PeTTF26/LOC_Os05g48690
	PeTTF29/LOC_Os02g33770
	PeTTF34/LOC_Os10g37240

### Gene structure and conserved motifs in Moso bamboo

**Fig. 3:**
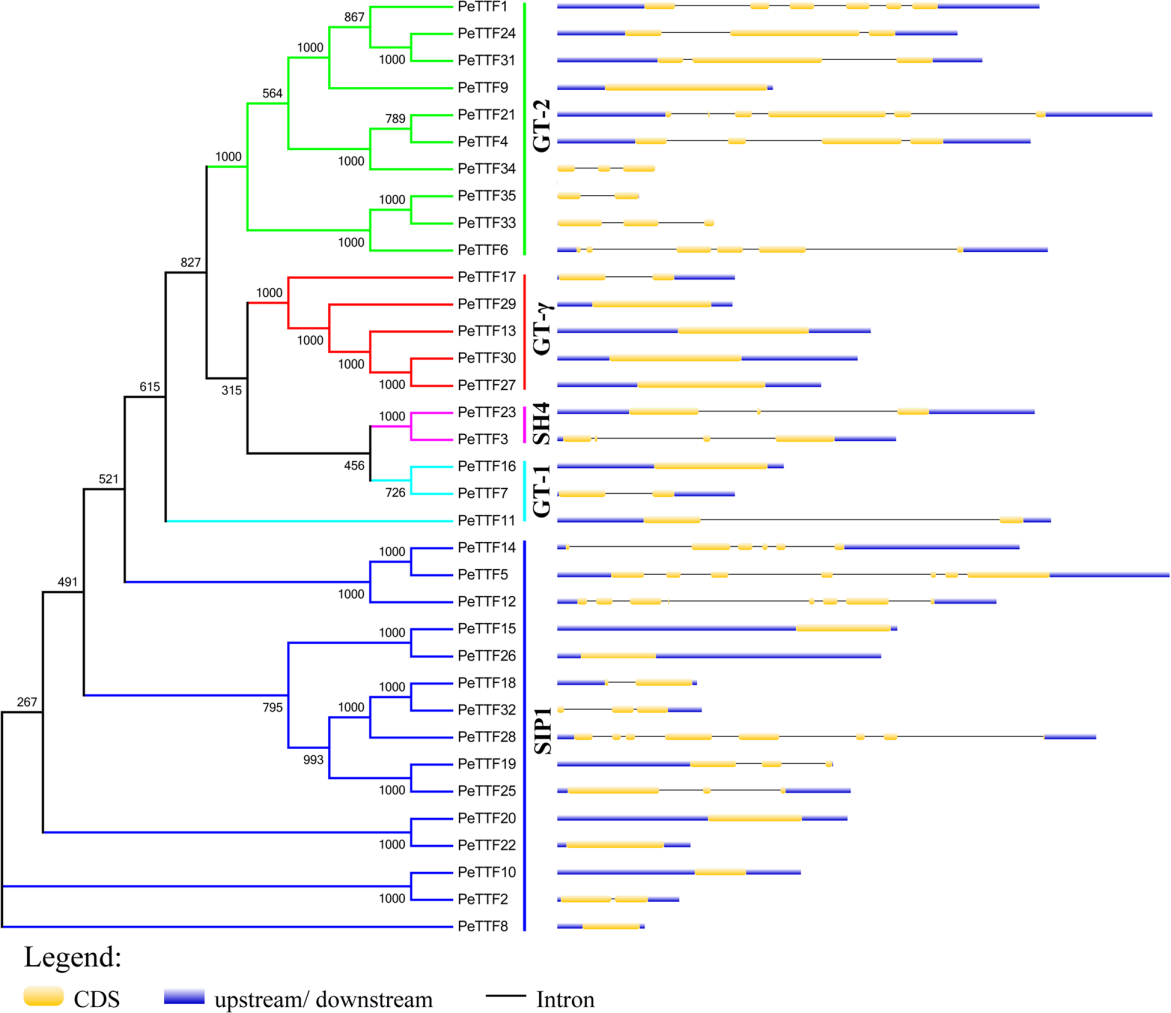
Phylogenetic relationships and gene structures of TTF genes in Moso bamboo. Left: Based on the results of sequence alignment, using the NJ method to construct the Phylogenetic tree of TTFs. Five different colors represented different subfamilies. Right: Exons, introns and untranslated regions (UTRs) are indicated by yellow rectangles, gray lines and blue rectangles, respectively

Gene family evolution depends on the diversification of gene structures. The coding sequence of individual TTF genes in Moso bamboo were analyzed, and the similarity/difference among different subfamilies was analyzed according to exon/intron structure and evolutionary tree (Fig. 3). Similar exon/intron structures were observed in closely related genes of the same subfamily, with *PeTTF13*, *− 27*, *− 29* and *− 30* of the *GT-γ* subfamily containing only one exon. The results showed that 34% of the *PeTTF* genes (*PeTTF8*, *− 9*, *− 10*, *− 13*, *− 15*, *− 16*, *− 20*, *− 22*, *− 26*, *− 27*, *− 29*, *− 30* and *− 37*) had no introns, while the remaining genes contained 1–7 introns. To better understand the exon/intron structures of the paralogous genes, we further analyzed the 12 pairs of paralogous genes. Among these genes, five pairs (*PeTTF15/− 26*, *PeTTF19/− 25*, *PeTTF20/− 22*, *PeTTF24/− 31* and *PeTTF33/− 35*) showed the same number of exons. These results may indicate that these genes have similar functions.

**Fig. 4:**
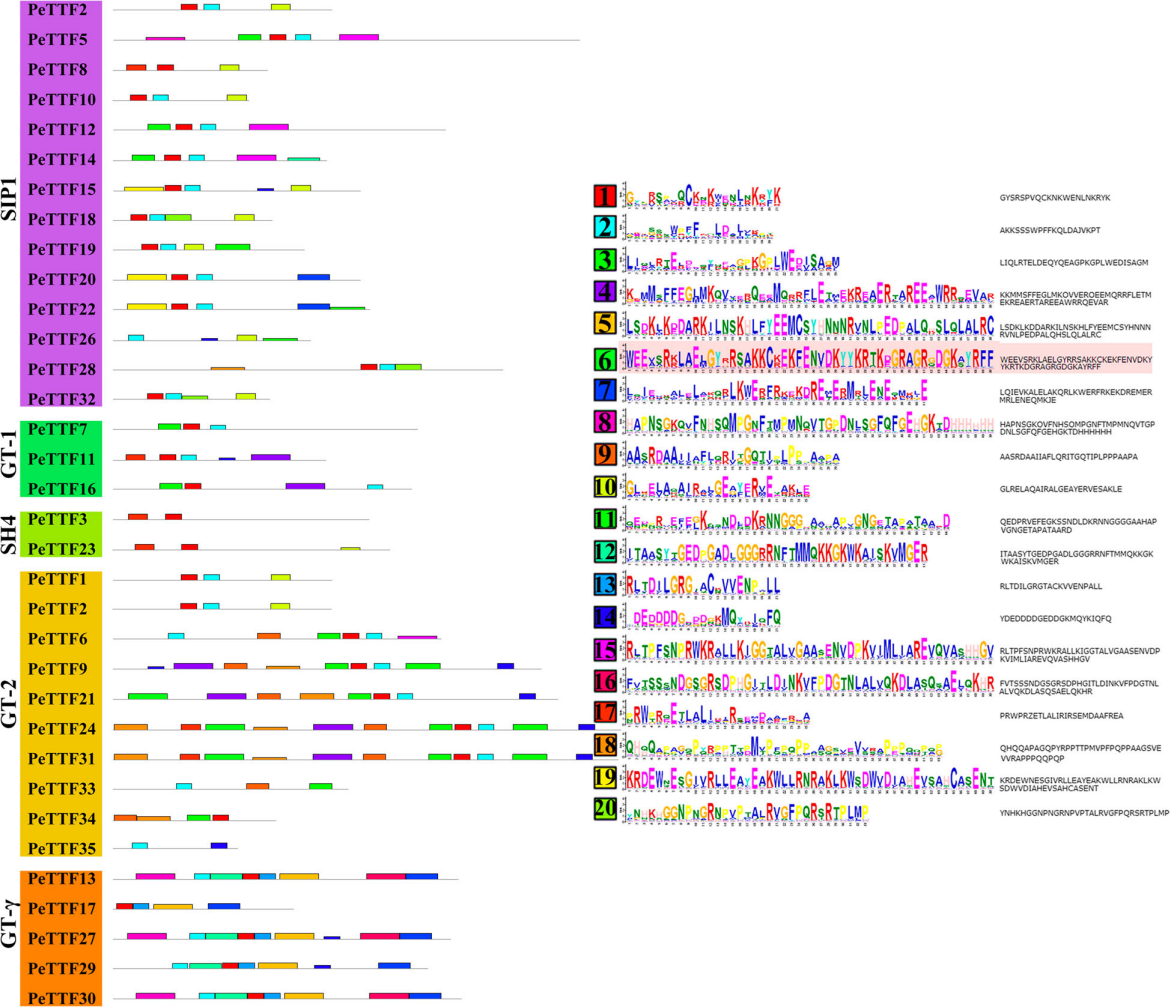
Schematic representation of 20 conserved motifs in the TTF genes. Using the online MEME program, the conserved motifs in the TTF genes were identified. Different colored boxes represented different specific motifs. The length of each box in the figure does not represent the actual motif size

The diversity of the *TTF* gene family was further examined using the MEME motif search tool (http://meme-suite.org/tools/meme). Twenty motifs were identified, the specific information for which is shown in Additional file 2: Table S2. As shown in Fig. 4, some subfamilies contained less than five motifs (the *SH4*, *GT-1* and *SIP1* subfamilies). Almost all *TTF* genes contained Motif 1 and Motif 2. All members of the *GT-γ* and *SIP1* subfamilies had similar motifs (Fig. 4), indicating that the PeTTF protein sequences were highly conserved within subfamilies.

### Strong purifying selection in Moso bamboo

To further explore the evolutionary patterns and divergence of the TTF gene family in Moso bamboo, 12 paralogous pairs of Moso bamboo genes were identified. Then, the timing of duplication events was calculated using the formula T = Ks/2λ [55]. The average Ks values and estimated time for each duplication event among the *TTF* genes of Moso bamboo are listed in Table 3. According to the Ks values, the 12 pairs of duplicate genes were divided into the following groups. The first group included *PeTTF7/− 16* and *PeTTF19/− 25*. These two paralogous pairs had similar Ks values, with an average value of about 1.26, and the corresponding time of the duplication events was about 97 Mya. The second group included *PeTTF3/− 23*, *PeTTF15/− 26* and *PeTTF33/− 35*. The Ks values of these three paralogous pairs were similar, with an average of about 0.63, and the corresponding time of the duplication events was about 48.12 Mya. The Ks values of the remaining seven pairs were similar to the average value of 0.21, corresponding to duplication events approximately 16.28 Mya. In addition, only three paralogous pairs had duplication events that occurred earlier than the divergence of Moso bamboo (12 Mya), indicating that most of the TTF gene duplication events occurred earlier than the speciation of Moso bamboo.

**Table 3:** Ka/Ks analysis and estimated divergence time of *PeTTF* genes

Duplicated *TTF* gene pairs	Ka	Ks	Ka/Ks	Duplication data (MY)
PeTTF2–10	0.19628	0.28456	0.6898	21.89
PeTTF3–23	0.3001	0.57003	0.5265	43.85
PeTTF4–21	0.40385	0.30886	1.3076	23.76
PeTTF5–14	0.24701	0.26566	0.9298	20.44
PeTTF7–16	2.05006	1.33589	1.5346	102.76
PeTTF15–26	0.37904	0.7751	0.4890	59.62
PeTTF18–32	0.22935	0.26615	0.8617	20.47
PeTTF19–25	2.3437	1.19812	1.9561	92.16
PeTTF20–22	0.03489	0.15364	0.2271	11.82
PeTTF24–31	0.06509	0.10698	0.6084	8.22
PeTTF27–30	0.03282	0.0955	0.3437	7.35
PeTTF33–35	1.26582	0.53139	2.3821	40.88

We also calculated the pairwise Ka (non-synonymous)/Ks ratios of repeated non-*TTF* genes (flanking genes) between repeated regions containing *TTF*s in Moso bamboo. A previous study indicated that a Ka/Ks ratio = 1 suggests neutral selection, while a Ka/Ks ratio > 1 indicates positive selection and a Ka/Ks ratio < 1 indicates negative selection or genetic purification (Additional file 7: Table S5). To investigate the selection pressure during the evolution of the *PeTTF* family, Ka and Ks replacement rates for each duplicated pair of *PeTTF* genes were calculated (Table 3). These results showed that the Ka/Ks rates of four of the 12 pairs of paralogous genes were greater than 1, suggesting that a small number of *TTF* genes had undergone positive selection, which can indicate the production of new genes. The other paralogous genes had undergone strong negative selection, indicating that most *TTF* genes are evolving more slowly.

Additionally, we performed a sliding-window analysis of the *PeTTF* gene fragments to better investigate the Ka/Ks ratios of different loci in the coding sequence (Additional file 3: Figure S1). These results indicated that the TTF gene family had undergone strong purifying selection (Ka/Ks < < 1) during the process of evolution.

### Expression profile analysis of *TTF* gene family

**Fig. 5:**
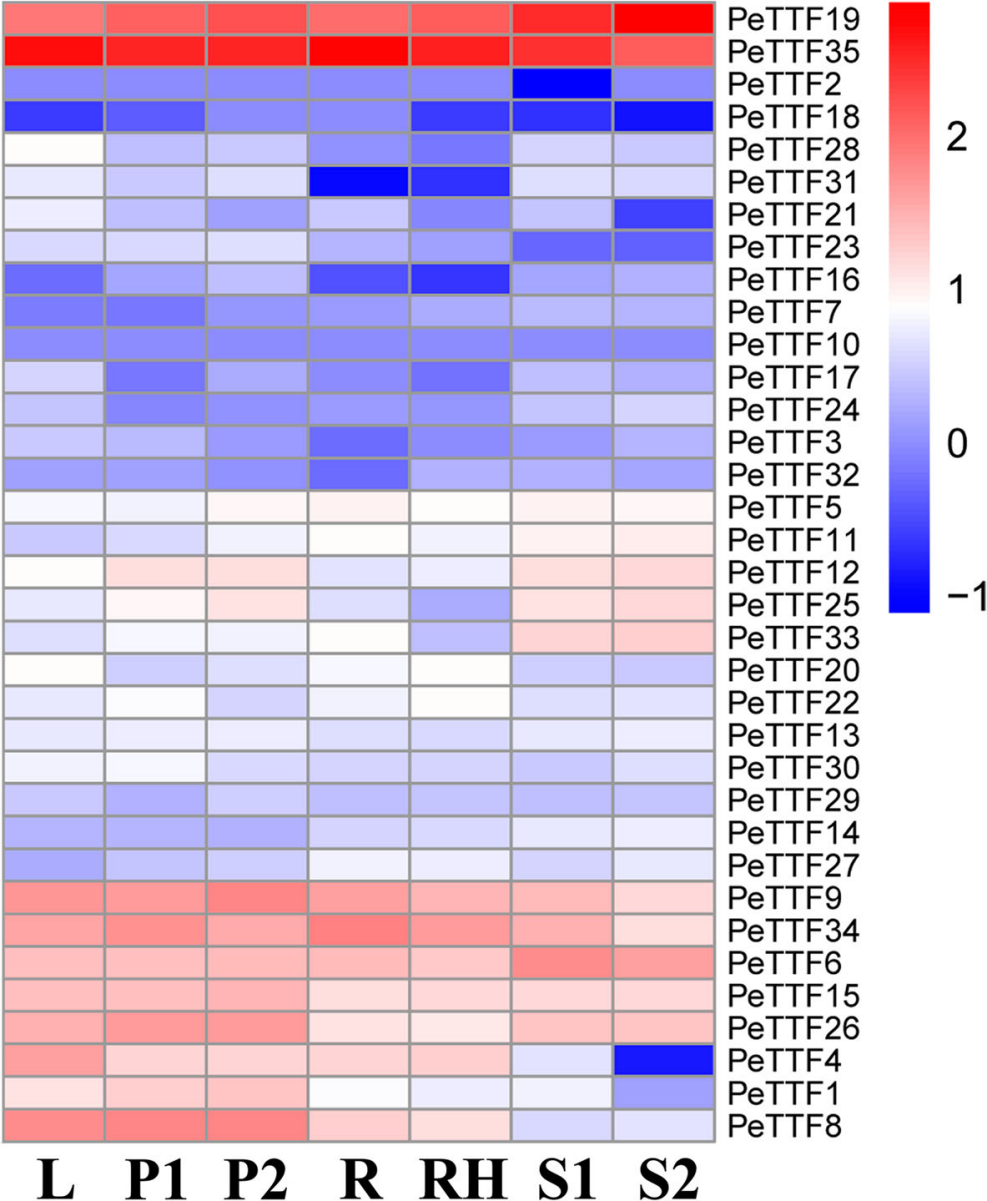
Expression profiles of poplar TTF genes in different tissues and developmental stages. Heatmap shows the hierarchical clustering of the 35 PeTTF genes among the different tissues. The abbreviation represents specific developmental stages: leaf (L); early panicle (P1); advanced panicle (P2); root (R); rhizome (RH); 20 cm shoot (S1); 50 cm shoot (S2)

Data for the 35 TTF genes was obtained from the NCBI short read archive (SRA) database (https://trace.ncbi.nlm.nih.gov/Traces/sra/?study=ERP001341). In the data, most of the Moso bamboo *TTF* genes showed tissue-specific expression patterns, with 9, 10 and 11 genes expressed highly in the leaf (L), early panicle (P1) and advanced panicle (P2), respectively, suggesting that they might play important roles in plant growth. Two genes (*PeTTF19* and *PeTTF35*) were highly expressed in the leaf, early panicle, advanced panicle, root, rhizome, 20 cm shoot and 50 cm shoot. Most paralogous genes had similar expression patterns, such as *PeTTF19/− 25* and *PeTTF33/− 35* (Fig. 5). However, the duplication of genes potentially results in differences in expression patterns, revealing their different evolutionary fates. Here, for example, *PeTTF19* was highly expressed in the leaf (L), early panicle (P1), advanced panicle (P2), root (R), rhizome (RH), 20 cm shoot (S1) and 50 cm shoot (S2), while *PeTTF25* expression was comparatively low in those tissues.

**Fig. 6:**
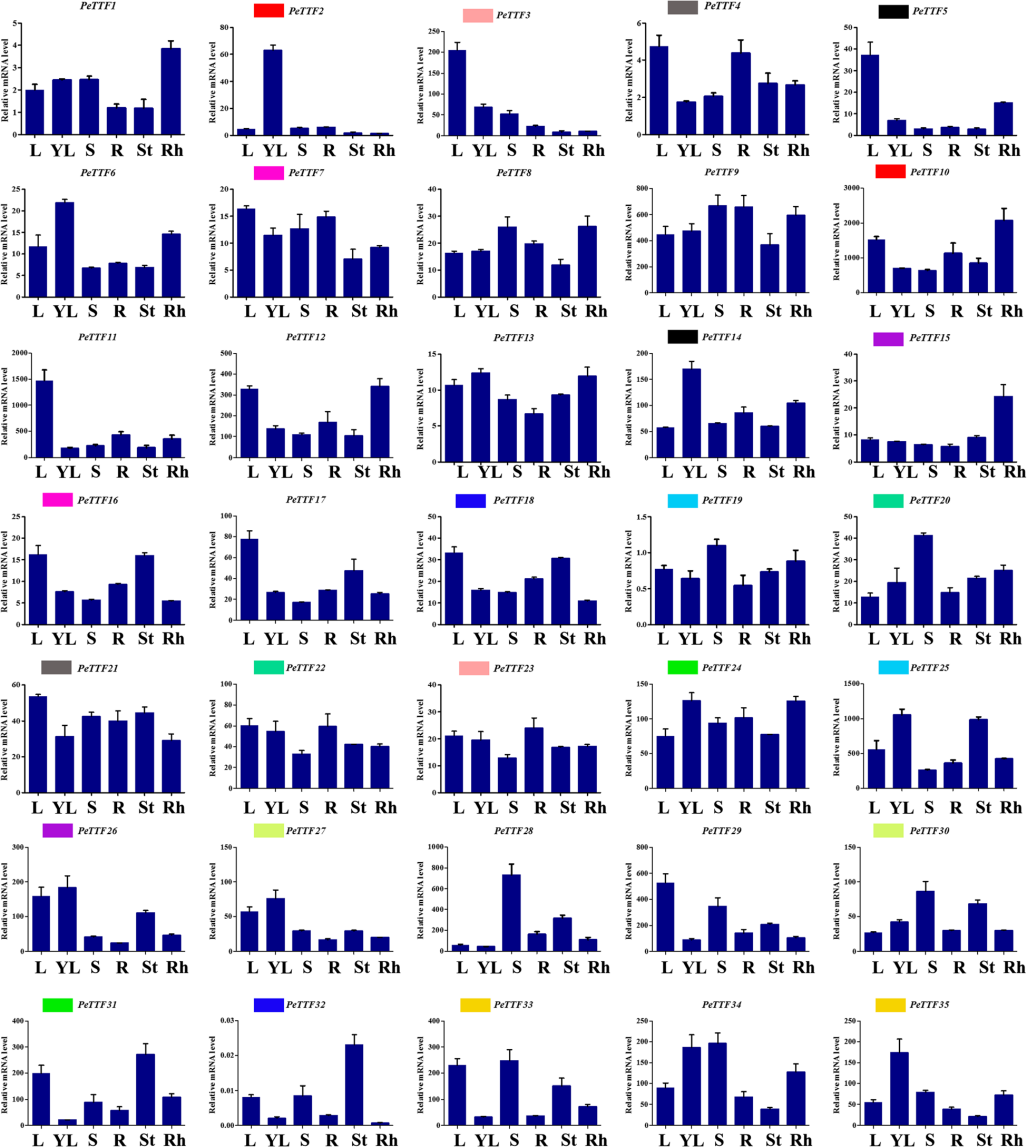
Expression profiles of poplar TTF genes across different tissues and developmental stages as revealed by qRT-PCR. Y-axis: relative expression levels; X-axis: the time course of stress treatments; Error bars, 6 ± SE. The abbreviation represents specific developmental stages: leaf (L); young leaf (YL); root (R); rhizome (RH); shoot (S); stem (St)

To obtain an overview of the *TTF* gene expression profiles, the expression patterns in six tissues, including young leaves, roots, rhizomes, stems, shoots and leaves, were studied by quantitative real-time PCR (qRT-PCR). This showed that the expression of most *PeTTF* genes was significantly higher in leaves (28.6%), young leaves (25.7%) and shoots (28.6%) (Fig. 6). According to the comparative analysis of paralogous genes, two pairs of paralogous genes showed similar expression patterns in different tissues (*PeTTF4/− 21* and *PeTTF7/− 16*). In contrast, the remaining 10 paralogous pairs (*PeTTF2/− 10*, *PeTTF3/− 23*, *PeTTF5/− 14*, *PeTTF15/− 26*, *PeTTF18/− 32*, *PeTTF19/− 25*, *PeTTF20/− 22*, *PeTTF24/− 31*, *PeTTF27/− 30* and *PeTTF33/− 35*) showed different expression in different tissues.

### Promoter analysis of *TTF* genes in Moso bamboo

**Fig. 7:**
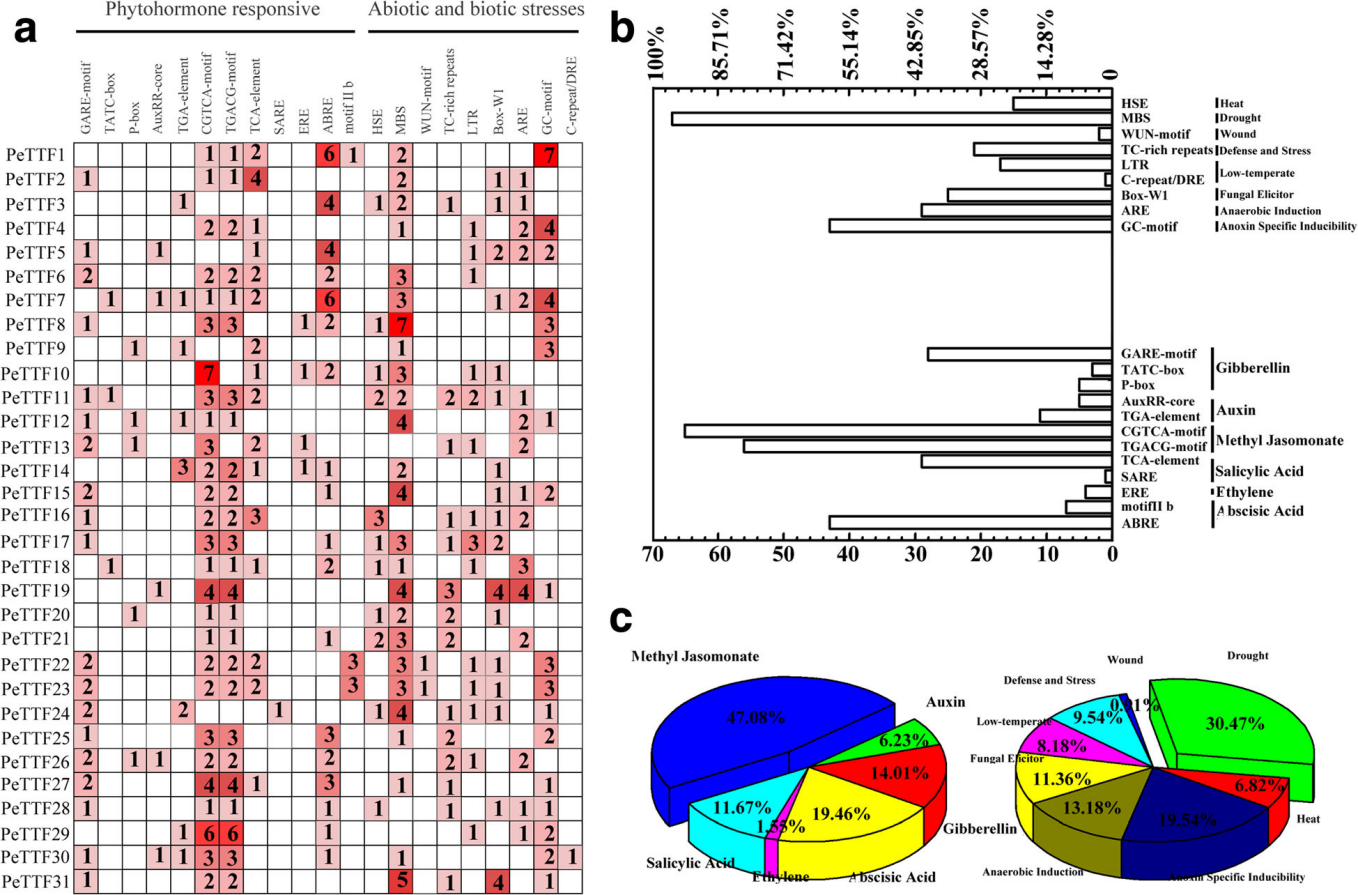
Cis-acting element analysis of the promoter regions of PeTTF genes. **a** Number of each cis-acting element in the promoter regions of PeTTF genes. **b** and **c** Statistics for the total number of PeTTF genes involved in different processes. Based on the functional annotation, the cis-acting elements were classified into two major classes: phytohormone responsive and abiotic and biotic stress-related cis-acting elements

To examine the *TTF* gene expression regulatory mechanisms, the DNA sequences 2000 bp upstream/downstream of the *TTF* genes were queried against the PlantCARE database. The *TTF* genes contained four types of cis-elements. The first was phytohormone responsive elements such as the GARE-motif, TATC-box, P-box, TGACG-motif, TCA-element and ABRE. The cis-acting regulatory elements involved in responses to methyl jasmonic acid (MeJA, 47.08%) were the CGTCA-motif (25.29%) and TGACG-motif (21.79%). The cis-acting regulatory elements involved in salicylic acid (SA, 11.67%) responses included the TCA-element (11.28%) and SARE (0.39%). SA and MeJA both play key roles in plant defense signaling [56, 57]. Thus, some *TTF* genes may be involved in pathogen resistance. The cis-acting regulatory elements involved in abscisic acid (ABA, 19.46%) responses included motif II b (2.72%) and the ABRE (16.74%). Gibberellin (GA, 14.01%) regulatory elements included the GARE-motif (10.89%), TATC-box (1.17%) and P-box (1.95%). Auxin (IAA, 6.23%) responsive cis-acting regulatory elements included the AuxRR-core (1.95%) and TGA-element (4.28%). ABA, GA and IAA all play major roles in plant growth and survival. Therefore, they may regulate the expression of some TTF genes (Fig. 7). The second type of cis-element was abiotic and biotic stress responsive elements, which included heat stress responsive (HSE, 6.82%), defense and stress responsive (TC-rich repeats, 9.54%), anaerobic-induced (ARE, 13.18%), anoxia-specific inducible (GC-motif, 19.54%) and drought-inducible (MBS, 30.47%) elements (Fig. 7). In addition to these, other phytohormone responsive (e.g. ERE) and abiotic and biotic stress responsive (e.g. wound and low-temperature) elements were found in the *TTF* gene family (Fig. 7). The third class was plant growth and development elements, including endosperm expression cis-acting regulatory elements (Skn-1_motif and GCN4_motif), a cis-acting regulatory element related to meristem-specific activation (CCGTCC-box) and circadian control cis-acting elements (circadian), which were highly represented in the *PeTTF* genes. The final class consisted of light responsive elements; for example, the G-box, GAG-motif and GT1-motif (Additional file 4: Table S3).

### Gene ontology (GO) annotation analysis and subcellular localization prediction

The association of 35 TTF genes with different biological processes was analyzed by GO annotation. In the molecular function category (Additional file 5: Table S4), six genes (*PeTTF1*, *− 7*, *− 9*, *− 21*, *− 24* and *− 31*) were annotated with the term ‘chromatin binding’ (Additional file 5: Table S4), but only three genes (*PeTTF3*, *− 7* and *− 23*) were annotated with ‘DNA binding’. In the biological process category, three genes (*PeTTF5*, *− 12* and *− 14*) were associated with cellular amino acid biosynthetic processes.

**Fig. 8:**
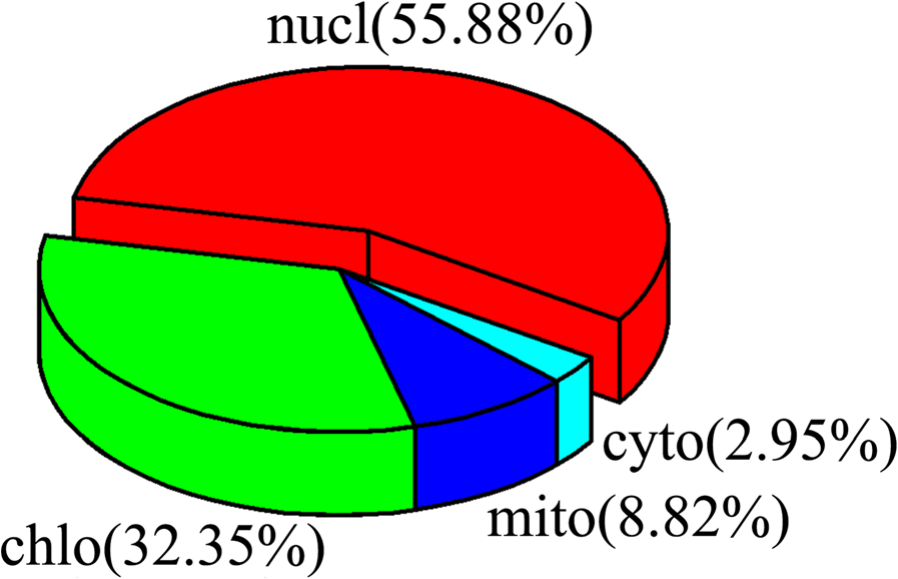
Subcellular localization of the TTF genes in Moso bamboo. Different colors represent different positions. Nucl, nucleus; chlo, chloroplast; cyto, cytoplasm; mito, mitochondrion

Subcellular localization analysis of the *TTF* gene products was also performed (Additional file 7: Table S5) (Fig. 8). Nineteen gene products were located in the nucleus (55.88%, nucleus), 11 were localized to chloroplasts (31.43%, chloroplast), three were situated in the mitochondria (8.56%, mitochondria), and only one was localized to the cytosol (2.86%, cytosol).

### Expression analysis of Moso bamboo *TTF* genes by qRT-PCR

**Fig. 9:**
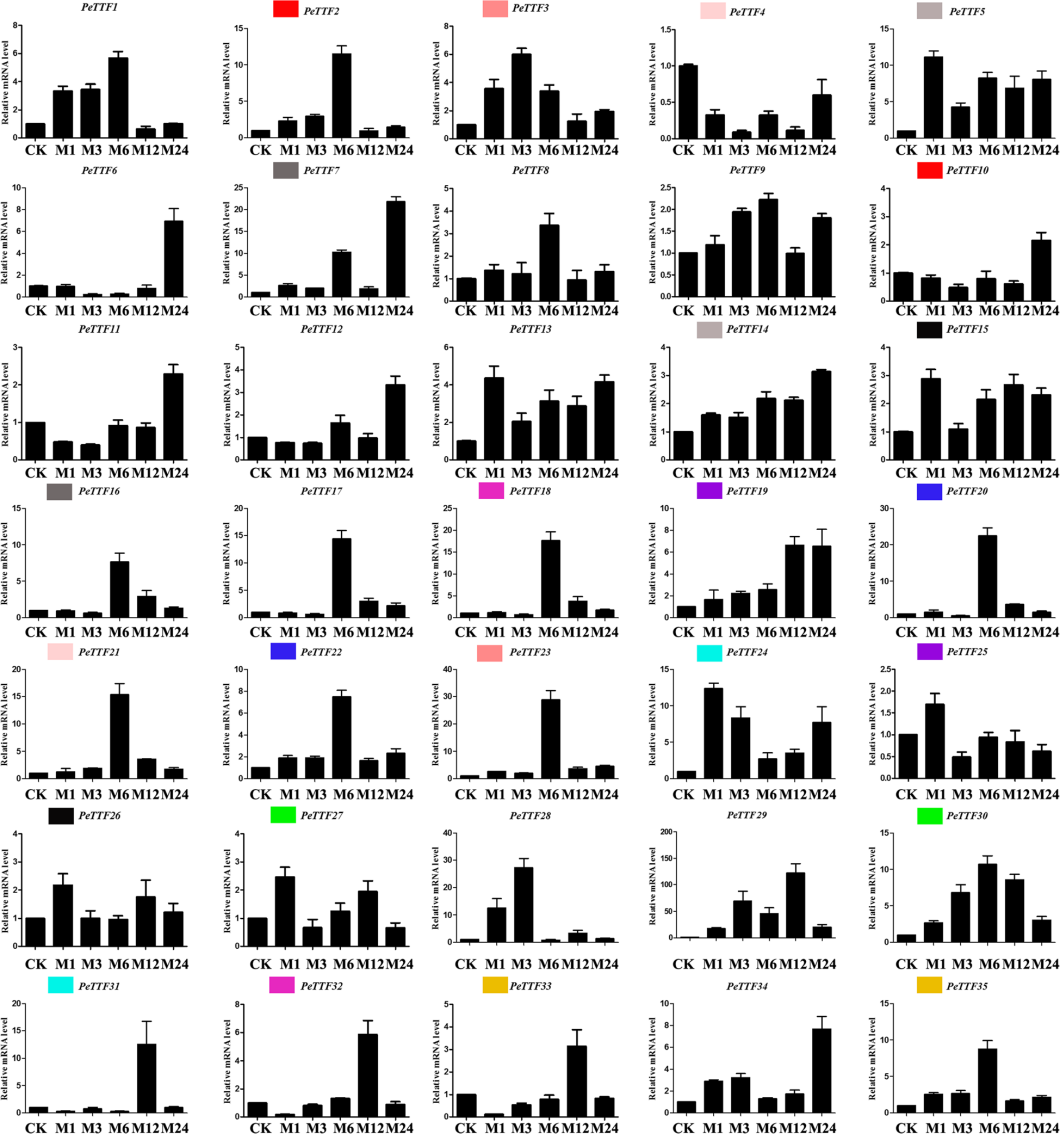
Expression patterns of all selected genes in Moso bamboo under MeJA treatment, as revealed by qRT-PCR. Y-axis: relative expression levels; X-axis: the time course of stress treatments; Error bars, 6 ± SE

During plant growth, various adverse environmental effects such as drought, disease and insect predation can influence plant development. Many stress-related genes can help manage responses to these adverse conditions. Primers for qRT-PCR were designed for the *TTF* genes and are listed in Additional file 6: Table S6. As the expression of *TTF* genes has previously been shown to be sensitive to MeJA treatment [12], qRT-PCR was used to examine the expression patterns of the 35 *TTF* genes in response to MeJA treatment in Moso bamboo. Most of the *PeTTF* genes were affected by MeJA but *PeTTF29* had the highest expression at 12 h (> 10-fold) (Fig. 9). In total, 17 genes were upregulated, while the *PeTTF4* gene was downregulated at all times. In the early stages, at 1 h after treatment, *PeTTF5*, *− 13* and *− 24* showed peak expression levels (> 10-fold, 4-fold and 24-fold, respectively). *PeTTF1*, *− 2*, *− 8*, *− 16*, *− 17*, *− 18*, *− 19*, *− 20*, *− 21*, *− 22*, *− 23*, *− 30* and *− 35* showed their highest expression levels after 6 h of MeJA treatment. Among these genes, the expression levels of *PeTTF20* and *− 23* were more than 20 times that of the control. *PeTTF3* and *− 28* were expressed relatively highly after 3 h of treatment (> 4-fold and 20-fold, respectively) and peak values for *PeTTF6*, *− 7*, *− 12*, *− 14* and *− 34* were observed after 24 h of treatment (all were upregulate at least three-fold) (Fig. 9). Furthermore, most paralogous pairs showed similar expression patterns; for example, *PeTTF15* and *− 26* were both upregulated, with their highest expression levels at 1 h (> 2-fold). As shown in Fig. 9, the expression patterns of *PeTTF4* and *− 21* were reversed.

**Fig. 10:**
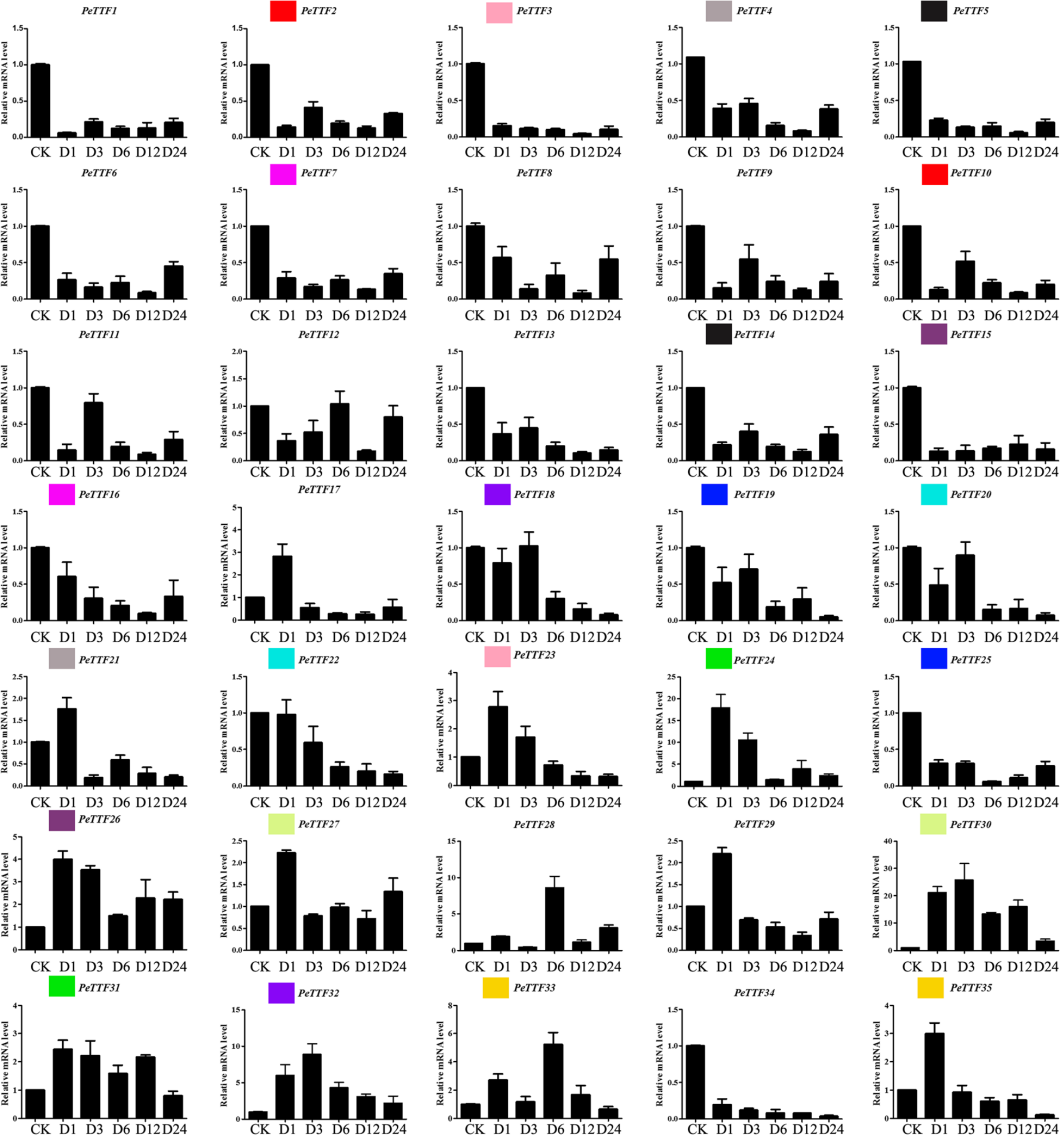
Expression patterns of all selected genes in Moso bamboo under PEG treatment, as revealed by qRT-PCR. Y-axis: relative expression levels; X-axis: the time course of stress treatments; Error bars, 6 ± SE

We also treated Moso bamboo seedlings with 20% PEG6000 solution and 200 mM NaCl to simulate drought and salt conditions, and observed *TTF* gene expression. Five genes (*PeTTF24*, *− 26*, *− 30*, *− 31* and *− 32*) were upregulated in response to PEG (drought) treatment while the other genes were downregulated (Fig. 10). High expression of *PeTTF17*, *− 23*, *− 24*, *− 26*, *− 27*, *− 29*, *− 31* and *− 35* was observed during the early stages of treatment (1 h), but only *PeTTF24* expression was more than 15 times the control value; the expression of the others was more than 2-fold. At 3 h, *PeTTF30* and *PeTTF32* had the highest expression (> 20-fold and 5-fold, respectively). *PeTTF28* and *PeTTF33* had the highest expression at 6 h (> 4-fold). *PeTTF24*, *− 28*, *− 30*, *− 32*, and *− 33* were also strongly upregulated (> 4-fold) (Fig. 10).

**Fig. 11:**
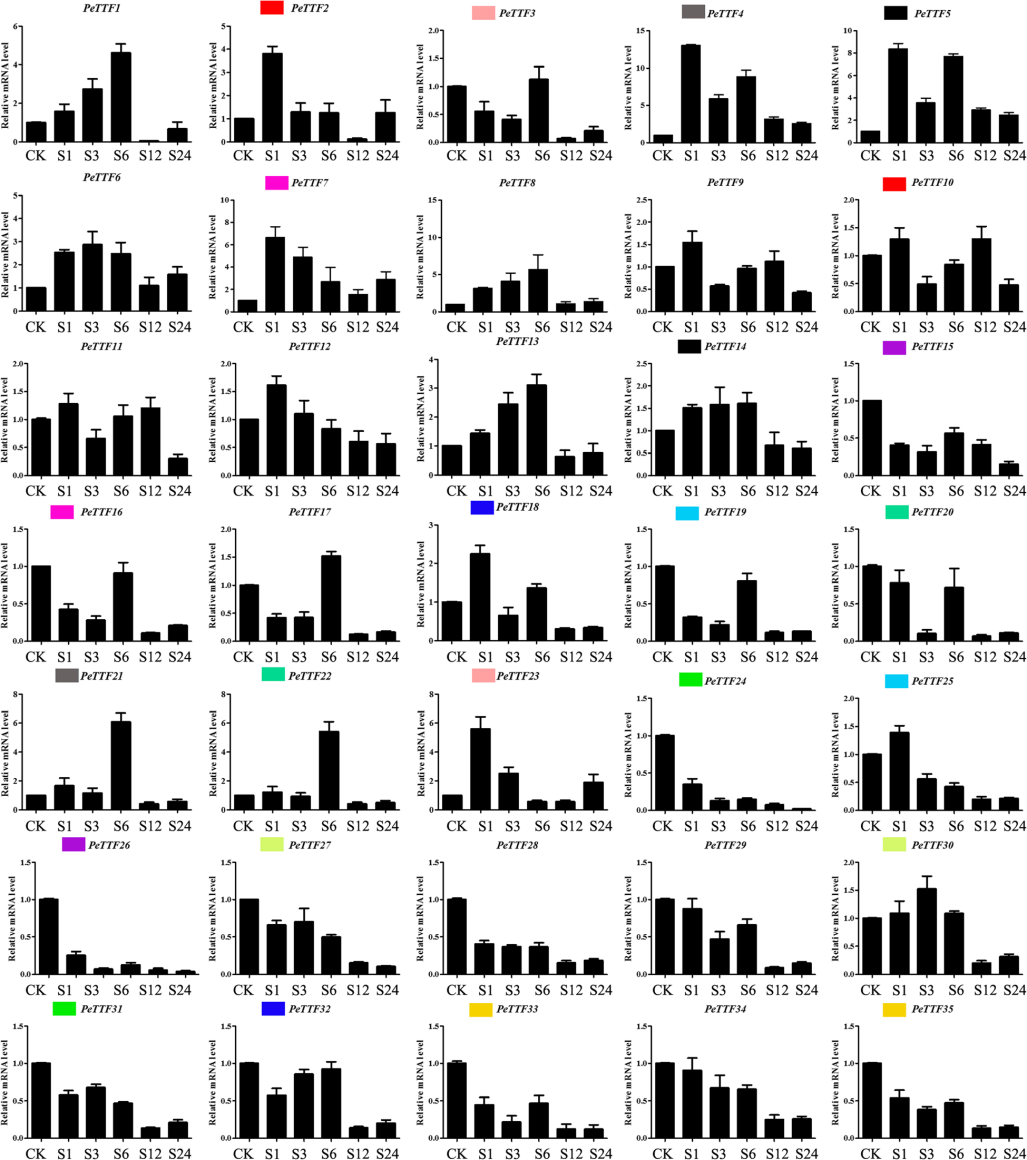
Expression patterns of all selected genes in Moso bamboo under salt treatment, as revealed by qRT-PCR. Y-axis: relative expression levels; X-axis: the time course of stress treatments; Error bars, 6 ± SE

In the salt treatment, only five genes (*PeTTF4*, *− 5*, *− 6*, *− 7* and *− 9*) were upregulated, but some genes were upregulated in specific periods; for example, *PeTTF2* was upregulated from 0 to 6 h and downregulated from 6 to 12 h (Fig. 11). The expression of 11 genes (*PeTTF2*, *− 4*, *− 5*, *− 7*, *− 9*, *− 10*, *− 11*, *− 12*, *− 18*, *− 23* and *− 25*) peaked 1 h after treatment, among which *PeTTF2*, *− 4*, *− 5*, *− 7*, and *− 23* were highly expressed (> 3-fold) (Fig. 11). These results suggested that some *PeTTF* genes play significant roles in the regulation of insect injury, drought and salt stress responses (Fig. 11).

### Subcellular localization of *PeTTF29*

**Fig. 12:**
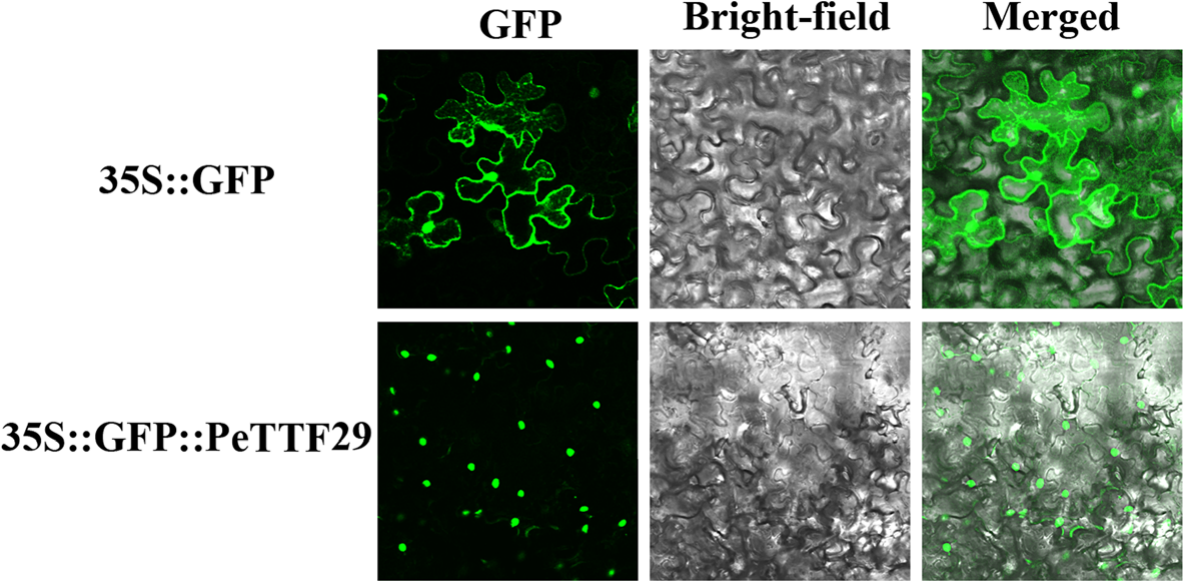
Nuclear localization of PeTTF29. The 35S::GFP::PeTTF29 construct and the control vector 1305(35S::GFP) were transformed into *Nicotiana tabacum* leaves. The GFP signals in root cells were observed by confocal microscopy

*PeTTF29* was predicted to be located in the nucleus by subcellular localization analysis. To confirm this prediction, a *PeTTF29-GFP* expression vector was transformed into tobacco. In the transgenic plants, the *GFP* signal was observed only in the nucleus (Fig. 12), whereas the plants transformed with constitutively expressed *GFP* control vectors show green fluorescence throughout the cells. This results indicated that *PeTTF29* was a nuclear protein.

### Transcription activation assay of *PeTTF29*

**Fig. 13:**
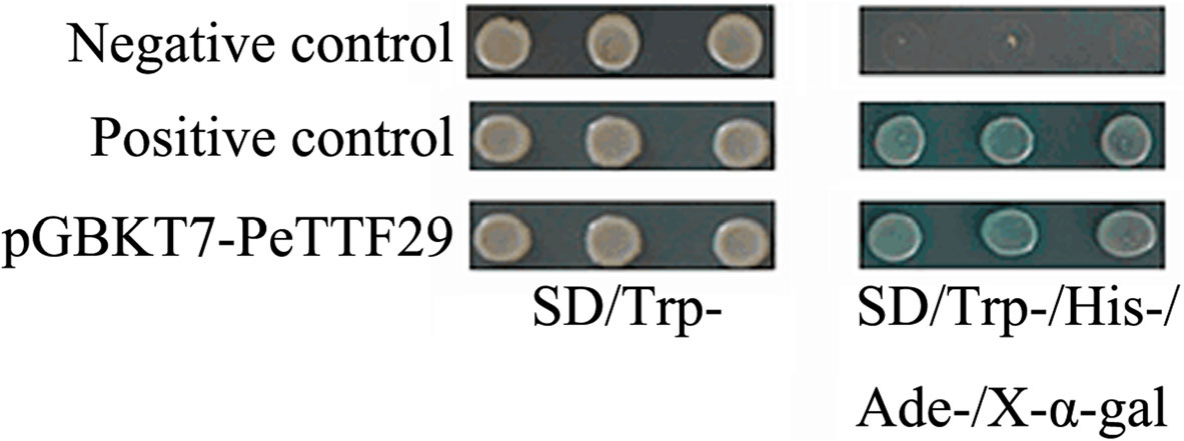
Transcriptional activity analysis of PeTTF29. Positive control, pGBKT7–53 and pGBKT7-T; Negative control, pGBKT7

The yeast yeast fusion expression vector *PeTTF29-pGBKT7* was constructed and transformed into yeast cells to detect transcriptional activity. Yeast cells containing the full-length *PeTTF29* fragment grew well on the *SD/Trp* − plate and were also able to grow well on the *SD/Trp−/His−/Ade−/X-α-gal* plate and produce a blue color reaction; the positive control showed the same results (Fig. 13). However, although the negative control was able to grow well on the *SD/Trp* − plate, it was unable to grow on the *SD/Trp−/His−/Ade−/X-α-gal* plate and did not produce a blue reaction. The above results indicated that *PeTTF29* was active in the yeast *H*_*2*_*GOLD*.

## Discussion

Previous studies of the *TTF* gene family have been reported in various plant species including Arabidopsis, rice, tomato, and chrysanthemum [3–7, 12, 13]. However, this family has never been examined in depth in Moso bamboo. Therefore, in the present study, we performed a genome-wide analysis of the *TTF* gene family in Moso bamboo and used qRT-PCR to investigate its antagonistic regulation.

In total, 35 *TTF* gene members were identified in Moso bamboo (Table 1). According to their phylogenetic relationships with TTF proteins from Arabidopsis and rice (Additional file 1: Table S1), these genes were divided into five subfamilies (SIP1, GT-1, SH4, GT-2, and GT-γ) (Fig. 1). The relatively short branches and intervals indicated that these proteins were highly conserved and that mutations were not common in their evolution, suggesting that they have similar functions. In addition, many *TTF* genes were clustered together at the ends of the branches, and high sequence similarity was observed between certain gene pairs. This also indicted that these genes may have similar functions.

The 35 genes contained varying numbers of exons and introns, indicating the diversity of the TTF gene family in Moso bamboo (Fig. 3). Nevertheless, in terms of the length of exons or the number of introns, the most closely related genes in the same subfamily shared similar gene structures. Some genes had unique exon/intron structures; for example, some Arabidopsis and rice genes had 16 or more introns [3, 6], while the Moso bamboo genes had substantially less introns (less than seven), indicating that the *PeTTF* genes may have lost introns or the rice and Arabidopsis genes have obtained introns. Twelve genes in Moso bamboo lacked introns, whereas a maximum of nine genes in Arabidopsis and rice had no introns, indicating that either Moso bamboo genes had lost exons or the rice and Arabidopsis genes had gained exons during the evolutionary process. Furthermore, MEME analysis showed that TTF protein-specific functions could be conferred by a specific sequence motif present in each subfamily. Overall, the similarity of the genetic structures and motif compositions of most *TTF* genes in each subfamily supported the phylogenetic analysis.

Genome duplication events can allow genes to evolve new functions, so they play a key role in increasing the genomic content and the diversification of gene functions. Moso bamboo (35 *PeTTF* genes) contained more *TTF* genes than rice (31 *OsTTF* genes) and Arabidopsis (28 *AtTTF* genes) [3, 6]. Moso bamboo has the largest genome among the three plants, as well as the largest number of chromosomes (2n = 48), which likely contributed to the greater number of *TTF* genes in Moso bamboo than in the other two species (as shown in Additional file 1: Table S1) [58]. In our analysis, we identified 12 duplicated gene pairs (Table 2) in Moso bamboo. The Ka/Ks ratios of eight pairs were less than 1 (Table 3), indicating that these genes had been subjected to purifying selection. We used a sliding Ka/Ks window to analyze each pair of *TTF* paralogs to further study the effects of purifying selection on the 12 paralogous pairs (Additional file 3: Figure S1). This clearly showed that strong purifying selection was experienced by the paralogous genes. Eight genes were under positive selection in particular regions, indicating that the *TTF* genes of Moso bamboo were also restrained in forward evolution to ensure their stability.

According to expression profile analysis, the 35 *TTF* genes are expressed in different tissues of Moso bamboo. Previous studies have suggested that *TTF* genes can regulate light-responsive genes [59, 60], and that the loss of the *AtGTL1* gene can affect the efficiency of water use by reducing the stomatal density [61], thereby increasing plant tolerance to water scarcity. These previous studies have shown relationships between *TTF* genes and leaf stomata or photoreactions. Accordingly, *PeTT19* and *− 35* were highly expressed in leaves, suggesting that (i) these two genes are associated with photosynthetic genes, or (ii) these genes are related to stomata or photosynthesis. Additionally, plant roots can quickly perceive changes in the soil and issue a series of signals to the branches and leaves to reduce root damage under drought conditions. In our research, *PeTTF35* was highly expressed in roots, indicating that this gene might strengthen the root organization to better absorb moisture and adapt to drought conditions. *TTF* genes expressed in different tissues with the ability to convey resistance could be used to improve plant tolerance to these stresses [62].

Promoter cis-elements play important roles in the biotic and abiotic stress responses of Moso bamboo. In this study, most promoter regions of the TTF genes were found to contain phytohormone and biotic and abiotic stress responsive cis-elements including the GARE-motif, TATC-box, P-box, and MBS. *PeTTF7* and *− 11* had 11 cis-elements, indicating they have important functions under different stresses. Notably, *PeTTF10* contained the most copies of the CGTCA-motif cis-element (7). However, it showed no significant expression change under MeJA treatment by qRT-PCR. This may be because the gene is upregulated after 24 h. *PeTTF27* had two MeJA stress-related cis-elements (the CGTCA-motif and TGACG-motif). The qRT-PCR results showed that *PeTTF27* was not only induced by SA, but also by ABA and MeJA, indicating that ABA and MeJA responses are closely related to SA responses.

Plants are often threatened by abiotic and biological stresses, which can cause severe damage or even be fatal. However, many genes have been genetically altered to help plants adapt to these stresses. Therefore, on the basis of the expression in different tissues (Fig. 5), seedling leaves were used to detect gene expression and perform qRT-PCR. Figure 5 shows that nine genes (*PeTTF4*, *− 6*, *− 8*, *− 9*, *− 15*, *− 19*, *− 26*, *− 34*, and *− 35*) were highly expressed whereas seven genes (*PeTTF4*, *− 6*, *− 8*, *− 9*, *− 15*, *− 19*, and *− 34*) were downregulated after PEG treatment, seven genes (*PeTTF6*, *9*, *− 15*, *− 19*, *− 26*, *− 34*, and *− 35*) were downregulated after salt treatment and four genes (*PeTTF4*, *− 6*, *− 9*, *− 15*, and *− 26*) were downregulated after MeJA treatment. In addition, six genes (*PeTTF23*, *− 24*, *− 28*, *− 30*, *− 32* and *− 33*) showed low in expression in Fig. 5, but the expression of these genes rapidly increased after PEG treatment. The expression of eight genes (*PeTTF1*, *− 2*, *− 5*, *− 7*, *− 13*, *− 21*, *− 22* and *− 23*) also began to increase rapidly after salt treatment and the expression of 20 genes (*PeTTF1*, *− 2*, *− 3*, *− 5*, *− 7*, *− 12*, *− 13*, *− 16*, *− 17*, *− 18*, *− 20*, *− 21*, *− 22*, *− 23*, *− 24*, *− 28*, *− 29*, *− 30*, *− 31*, and *− 32*) began to increase after MeJA treatment. From these results, it can be seen that Moso bamboo *TTF* genes play an important role in coping with different stresses.

Using qRT-PCR, we analyzed the transcription levels of *TTF* genes in response to biotic stress (MeJA) and abiotic stress (drought and salt). The results indicated that each *PeTTF* gene was differentially expressed after the MeJA, drought and salt treatments, which could provide a useful resource for future gene function analysis. Therefore, we used the GraphPad software to visualize the expression patterns of the *TTF* genes under different stress conditions (Fig. 12), which suggested the *TTF* gene family in Moso bamboo may play a key role in regulating abiotic/biological stress responses. In this family, similar sequences and expression patterns were observed between most duplicated genes, indicating that the regulatory sequences responsive to stress conditions had not, generally, diverged significantly through evolution following gene replication.

No previous studies on the functions of *TTF* gene members in Moso bamboo have been reported. Therefore, we screened a potential gene (*PeTTF29*) associated with abiotic stress in our experiments that was highly homologous to *LOC Os02g33770* in rice according to evolutionary analysis. We used Blast (NCBI) to compare the CDSs of these two genes and the results showed that their homology was 80% or more. *LOC Os02g33770* has been reported to be associated with abiotic stress responses [4], which suggests that *PeTTF29* may also be involved in some abiotic stress responses. To further explore the function of *PeTTF29*, we conducted subcellular localization and transcriptional activity experiments. These experiments showed that PeTTF29 was localized in the nucleus (Fig. 12) and had transcriptional activity (Fig. 13). Therefore, this gene had the basic properties that most transcription factors possess, indicating that it is a typical transcription factor gene.

## Conclusion

In summary, the structural diversity of Moso bamboo TTF proteins indicates they have diverse functions and may be associated with adaptation to different environmental stresses at different developmental stages. We analyzed 35 members of the *TTF* gene family in Moso bamboo, which were divided into five different subfamilies. Comparisons between the *TTF* genes in Moso bamboo and those in other model species confirmed their extensive homology and indicated when they had evolved. The *TTF* gene family was found to have expanded through large-scale gene duplication. These genes have continued to evolve at the protein level while being subjected to strong positive selection. The expression patterns of the 35 *TTF* genes were analyzed under two abiotic stresses (drought and salt) and one biotic stress (MeJA). Subcellular localization and transcriptional activity experiments showed that PeTTF29 is localized in the nucleus and has transcriptional activity. The results of this study will help to increase our understanding of the *TTF* family members, including their possible contributions to abiotic stress responses and other putative functions in Moso bamboo growth and development.

## Additional files

Additional file 1: **Table S1.** Detailed information about *TTF* genes in rice and *Arabidopsis*. (DOCX 25 kb)

Additional file 2: **Table S2.** MEME motif sequences and lengths of *TTF* gene family proteins in Moso bamboo. (DOCX 22 kb)

Additional file 3: **Figure S1.** Sliding window plots of the TTF genes in Moso bamboo. (TIF 603 kb)

Additional file 4: **Table S3.** Promoter analysis of TTF proteins in Moso bamboo. (XLS 50 kb)

Additional file 5: **Table S4.** Details of the Gene Ontology annotation. (DOCX 21 kb)

Additional file 6: **Table S6.** Design the qRT-PCR primers. (DOCX 25 kb)

Additional file 7: **Table S5.** Subcellular localization of TTF gene in Moso bamboo. (DOCX 21 kb)

## Abbreviations

aa: Amino acids
ABA: Abscisic acid
bp: Base pair
CDS: Coding sequence
Da: Dalton
GFP: Green fluorescent protein
Ka: Number of non-synonymous substitutions per non-synonymous site
Ks: Number of synonymous substitutions per synonymous site
MeJA: Methyl jasmonate
MW: Molecular weight
MYA: Million years ago
NJ: Neighbor-joining
PEG: Polyethylene glycol
Pi: Isoelectric point
qRT-PCR: Quantitative real-time PCR
SA: Salicylic acid
TTF: Trihelix transcription factors
