# Supplementary material for: The trihelix family of transcription factors: functional and evolutionary analysis in Moso bamboo (Phyllostachys edulis)
Source: BMC Plant Biol. 2019 Apr 25;19:154. doi: 10.1186/s12870-019-1744-8 (PMC6482567; doi:10.1186/s12870-019-1744-8)
Supplement: Supplementary file 1 — Table S1. Detailed information about TTF genes in rice and Arabidopsis. (DOCX 25 kb) [file 12870_2019_1744_MOESM1_ESM.docx]

**Table S1** Detailed information about *TTF* genes in rice and *Arabidopsis*

| Name | Gene Identifier | Chr | Location coordinates (5'- 3') | ORF length (bp) | Protein | | |  |  |
| --- | --- | --- | --- | --- | --- | --- | --- | --- | --- |
|  |  |  |  |  | Length (a.a.) | PI | Mol.Wt. (Da) | |  |
| LOC_Os01g21590 | LOC_Os01g21590 | 1 | 12091633-12093276 | 1647 | 548 | 6.8209 | 57857.6 | |  |
| LOC_Os01g48320 | LOC_Os01g48320 | 1 | 27690411-27692209 | 1002 | 333 | 10.0969 | 35919.3 | |  |
| LOC_Os01g52090 | LOC_Os01g52090 | 1 | 29951353-29952644 | 972 | 323 | 5.2783 | 35763.6 | |  |
| LOC_Os01g70230 | LOC_Os01g70230 | 1 | 40665090-40666542 | 900 | 299 | 8.0911 | 31551.3 | |  |
| LOC_Os02g01380 | LOC_Os02g01380 | 2 | 219577-221707 | 1116 | 371 | 5.1785 | 40503.2 | |  |
| LOC_Os02g07800 | LOC_Os02g07800 | 2 | 4083023-4086047 | 1311 | 436 | 4.1757 | 46239.9 | |  |
| LOC_Os02g31160 | LOC_Os02g31160 | 2 | 18658028-18661837 | 1128 | 375 | 4.8825 | 39550.6 | |  |
| LOC_Os02g33610 | LOC_Os02g33610 | 2 | 20005963-20013852 | 2652 | 883 | 9.1397 | 97367.7 | |  |
| LOC_Os02g33770 | LOC_Os02g33770 | 2 | 20147866-20150010 | 1236 | 411 | 6.7634 | 46666.8 | |  |
| LOC_Os02g35690 | LOC_Os02g35690 | 2 | 21449599-21451348 | 1263 | 420 | 6.525 | 44438.7 | |  |
| LOC_Os02g43300 | LOC_Os02g43300 | 2 | 26099235-26103142 | 1890 | 629 | 4.597 | 67740.1 | |  |
| LOC_Os02g57530 | LOC_Os02g57530 | 2 | 35251393-35256043 | 2514 | 837 | 7.8374 | 92619.9 | |  |
| LOC_Os03g02240 | LOC_Os03g02240 | 3 | 752024-756170 | 2181 | 726 | 7.1062 | 76494.9 | |  |
| LOC_Os03g18330 | LOC_Os03g18330 | 3 | 10274876-10275520 | 648 | 215 | 10.3133 | 23714 | |  |
| LOC_Os03g18340 | LOC_Os03g18340 | 3 | 10277042-10277809 | 771 | 256 | 10.3418 | 27438.1 | |  |
| LOC_Os03g46350 | LOC_Os03g46350 | 3 | 26216934-26218063 | 1041 | 346 | 6.5258 | 36835.9 | |  |
| LOC_Os04g32590 | LOC_Os04g32590 | 4 | 19613118-19615783 | 993 | 330 | 5.633 | 34882.6 | |  |
| LOC_Os04g33300 | LOC_Os04g33300 | 4 | 20138880-20141866 | 909 | 302 | 8.6074 | 31995.5 | |  |
| LOC_Os04g36790 | LOC_Os04g36790 | 4 | 22212579-22214515 | 1257 | 418 | 7.0386 | 43926.2 | |  |
| LOC_Os04g40930 | LOC_Os04g40930 | 4 | 24296450-24301211 | 1161 | 386 | 6.0253 | 41926.8 | |  |
| LOC_Os04g45750 | LOC_Os04g45750 | 4 | 27077367-27080595 | 1590 | 529 | 5.8914 | 57456.9 | |  |
| LOC_Os04g45940 | LOC_Os04g45940 | 4 | 27210157-27211268 | 621 | 206 | 10.7441 | 22366.5 | |  |
| LOC_Os04g51320 | LOC_Os04g51320 | 4 | 30393182-30395776 | 837 | 278 | 7.4606 | 32270.1 | |  |
| LOC_Os05g03740 | LOC_Os05g03740 | 5 | 1643448-1644746 | 1005 | 334 | 6.1586 | 36946.9 | |  |
| LOC_Os05g48690 | LOC_Os05g48690 | 5 | 27899055-27902500 | 1044 | 347 | 10.5017 | 37444.3 | |  |
| LOC_Os08g37810 | LOC_Os08g37810 | 8 | 23951363-23953011 | 951 | 316 | 7.708 | 35062.4 | |  |
| LOC_Os09g38570 | LOC_Os09g38570 | 9 | 22188092-22191842 | 1014 | 337 | 7.0543 | 36335.9 | |  |
| LOC_Os10g37240 | LOC_Os10g37240 | 10 | 19930005-19934782 | 2586 | 861 | 5.7061 | 88757 | |  |
| LOC_Os10g41460 | LOC_Os10g41460 | 10 | 22284367-22285920 | 1014 | 337 | 9.1903 | 35636 | |  |
| LOC_Os11g06410 | LOC_Os11g06410 | 11 | 3091535-3096323 | 1455 | 484 | 6.7117 | 55062.9 | |  |
| LOC_Os12g06640 | LOC_Os12g06640 | 12 | 3218983-3222907 | 1302 | 433 | 6.5865 | 48773.1 | |  |
|  |  |  |  |  |  |  |  | |  |
| Name | Gene Identifier | Chr | Location coordinates (5'- 3') | ORF length (bp) | Protein | | | | |
|  |  |  |  |  | Length (a.a.) | PI | Mol.Wt. (Da) | | Exons |
| AT1G13450 | AT1G13450 | 1 | 4612731-4615205 | 1221 | 406 | 6.8667 | 46675.9 | | 5 |
| AT1G21200 | AT1G21200 | 1 | 7421217-7423143 | 1332 | 443 | 6.287 | 50933.3 | | 1 |
| AT1G31310 | AT1G31310 | 1 | 11198353-11200140 | 1152 | 383 | 9.4992 | 42589.1 | | 2 |
| AT1G33240 | AT1G33240 | 1 | 12051471-12054546 | 2010 | 669 | 5.8532 | 74212.7 | | 3 |
| AT1G54060 | AT1G54060 | 1 | 20180679-20182324 | 1152 | 383 | 9.064 | 41731.6 | | 1 |
| AT1G76870 | AT1G76870 | 1 | 28857250-28858407 | 1158 | 385 | 7.0675 | 44957.2 | | 1 |
| AT1G76880 | AT1G76880 | 1 | 28865500-28868225 | 1812 | 603 | 6.6967 | 67879 | | 3 |
| AT2G33550 | AT2G33550 | 2 | 14210032-14211588 | 945 | 314 | 6.0421 | 34861.3 | | 3 |
| AT2G35640 | AT2G35640 | 2 | 14982835-14984182 | 1023 | 340 | 9.0497 | 38305.2 | | 2 |
| AT2G38250 | AT2G38250 | 2 | 16018357-16019500 | 870 | 289 | 7.0249 | 34307.4 | | 2 |
| AT2G44730 | AT2G44730 | 2 | 18437333-18438565 | 1119 | 372 | 9.4426 | 40781.8 | | 1 |
| AT3G10000 | AT3G10000 | 3 | 3076874-3078907 | 1446 | 481 | 7.9162 | 55502.6 | | 2 |
| AT3G10030 | AT3G10030 | 3 | 3092023-3094945 | 1629 | 542 | 6.558 | 59247.8 | | 7 |
| AT3G10040 | AT3G10040 | 3 | 3096415-3098071 | 1296 | 431 | 8.063 | 48870.3 | | 1 |
| AT3G11100 | AT3G11100 | 3 | 3476187-3477405 | 750 | 249 | 5.1728 | 28385.8 | | 2 |
| AT3G14180 | AT3G14180 | 3 | 4707113-4708848 | 1332 | 443 | 10.2708 | 48314.1 | | 1 |
| AT3G24490 | AT3G24490 | 3 | 8910770-8912196 | 1002 | 333 | 4.3696 | 39083 | | 1 |
| AT3G24860 | AT3G24860 | 3 | 9073623-9074682 | 933 | 310 | 9.7598 | 35191.4 | | 1 |
| AT3G25990 | AT3G25990 | 3 | 9504677-9506787 | 1119 | 372 | 5.7598 | 42782.9 | | 5 |
| AT3G54390 | AT3G54390 | 3 | 20137741-20139142 | 891 | 296 | 10.0601 | 33280.8 | | 2 |
| AT3G58630 | AT3G58630 | 3 | 21683568-21685941 | 966 | 321 | 9.9284 | 36046.4 | | 2 |
| AT4G31270 | AT4G31270 | 4 | 15183188-15184961 | 885 | 294 | 4.591 | 33369.4 | | 2 |
| AT5G01380 | AT5G01380 | 5 | 155639-157601 | 972 | 323 | 6.523 | 38272.7 | | 2 |
| AT5G03680 | AT5G03680 | 5 | 957744-961032 | 1776 | 591 | 7.0831 | 66638.8 | | 2 |
| AT5G05550 | AT5G05550 | 5 | 1639032-1640606 | 741 | 246 | 9.0214 | 28191.8 | | 2 |
| AT5G28300 | AT5G28300 | 5 | 10292651-10295283 | 1860 | 619 | 7.2368 | 71280.1 | | 2 |
| AT5G47660 | AT5G47660 | 5 | 19313008-19314636 | 1197 | 398 | 5.9406 | 45575.1 | | 2 |
| AT5G63420 | AT5G63420 | 5 | 25400386-25405968 | 2736 | 911 | 8.3897 | 100554 | | 17 |
|  |  |  |  |  |  |  |  | |  |
