# Supplementary material for: The trihelix family of transcription factors: functional and evolutionary analysis in Moso bamboo (Phyllostachys edulis)
Source: BMC Plant Biol. 2019 Apr 25;19:154. doi: 10.1186/s12870-019-1744-8 (PMC6482567; doi:10.1186/s12870-019-1744-8)
Supplement: Supplementary file 2 — Table S2. MEME motif sequences and lengths of TTF gene family proteins in Moso bamboo. (DOCX 22 kb) [file 12870_2019_1744_MOESM2_ESM.docx]

Table S2 MEME motif sequences and lengths of *TTF* gene family proteins in Moso bamboo

| Motif | Width | Best possible match |
| --- | --- | --- |
| 1 | 21 | GYSRSPVQCKNKWENLNKRYK |
| 2 | 20 | AKKSSSWPFFKQLDAJVKPT |
| 3 | 29 | LIQLRTELDEQYQEAGPKGPLWEDISAGM |
| 4 | 50 | KKMMSFFEGLMKQVVERQEEMQRRFLETMEKREAERTAREEAWRRQEVAR |
| 5 | 50 | LSDKLKDDARKILNSKHLFYEEMCSYHNNNRVNLPEDPALQHSLQLALRC |
| 6 | 50 | WEEVSRKLAELGYRRSAKKCKEKFENVDKYYKRTKDGRAGRGDGKAYRFF |
| 7 | 41 | LQIEVKALELAKQRLKWERFRKEKDREMERMRLENEQMKJE |
| 8 | 50 | HAPNSGKQVFNHSQMPGNFTMPMNQVTGPDNLSGFQFGEHGKTDHHHHHH |
| 9 | 29 | AASRDAAIIAFLQRITGQTIPLPPPAAPA |
| 10 | 25 | GLRELAQAIRALGEAYERVESAKLE |
| 11 | 44 | QEDPRVEFEGKSSNDLDKRNNGGGGAAHAPVGNGETAPATAARD |
| 12 | 41 | ITAASYTGEDPGADLGGGRRNFTMMQKKGKWKAISKVMGER |
| 13 | 21 | RLTDILGRGTACKVVENPALL |
| 14 | 21 | YDEDDDDGEDDGKMQYKIQFQ |
| 15 | 50 | RLTPFSNPRWKRALLKIGGTALVGAASENVDPKVIMLIAREVQVASHHGV |
| 16 | 50 | FVTSSSNDGSGRSDPHGITLDINKVFPDGTNLALVQKDLASQSAELQKHR |
| 17 | 25 | PRWPRZETLALIRIRSEMDAAFREA |
| 18 | 43 | QHQQAPAGQPYRPPTTPMVPFPQPPAAGSVEVVRAPPPQQPQP |
| 19 | 50 | KRDEWNESGIVRLLEAYEAKWLLRNRAKLKWSDWVDIAHEVSAHCASENT |
| 20 | 33 | YNHKHGGNPNGRNPVPTALRVGFPQRSRTPLMP |
|  |  |  |
