# Supplementary figures and images for: The trihelix family of transcription factors: functional and evolutionary analysis in Moso bamboo (Phyllostachys edulis)
Source: BMC Plant Biol. 2019 Apr 25;19:154. doi: 10.1186/s12870-019-1744-8 (PMC6482567; doi:10.1186/s12870-019-1744-8)

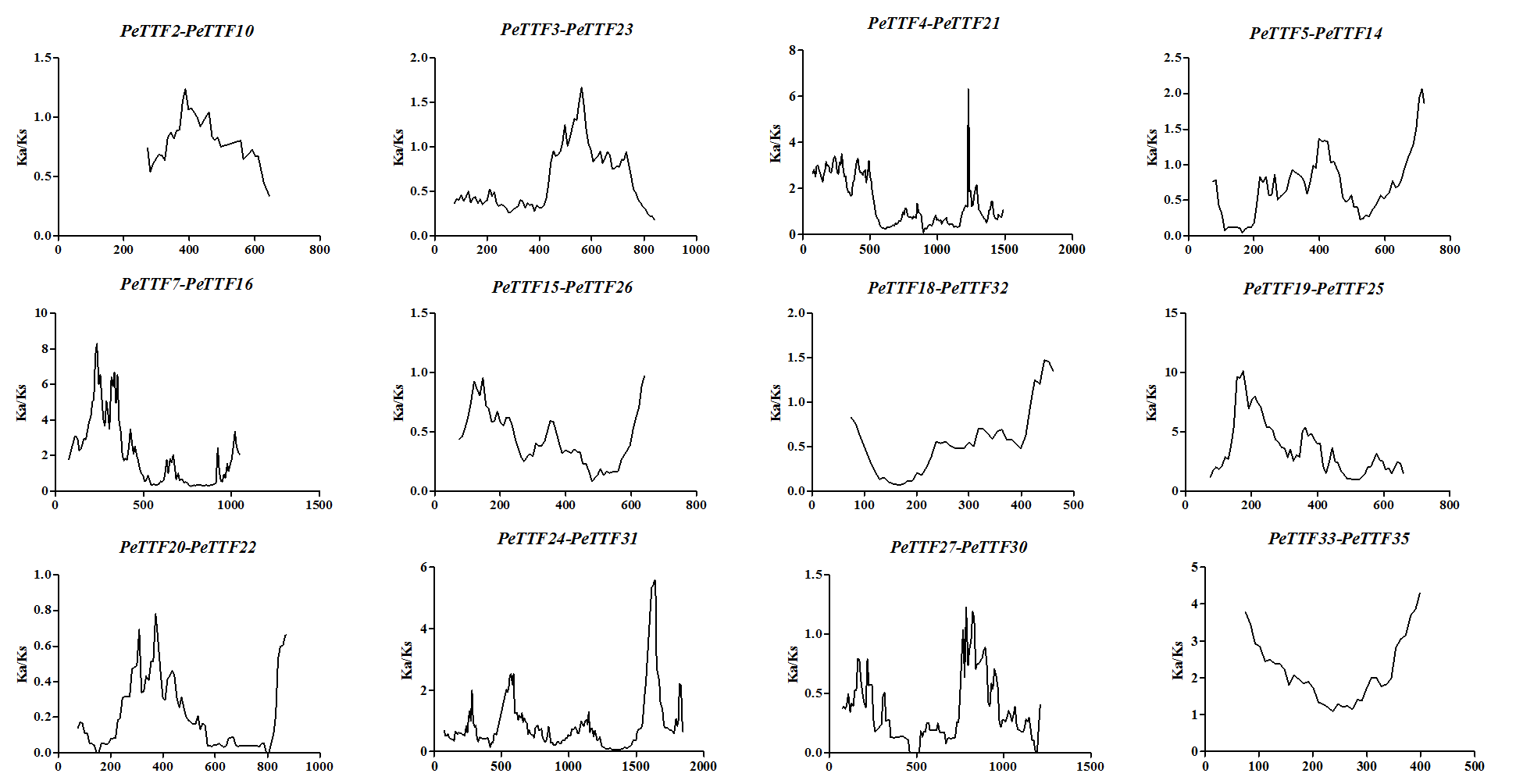

Supplement: Supplementary file 3 — Figure S1. Sliding window plots of the TTF genes in Moso bamboo. (TIF 603 kb) [file 12870_2019_1744_MOESM3_ESM.tif]
