# Supplementary material for: The trihelix family of transcription factors: functional and evolutionary analysis in Moso bamboo (Phyllostachys edulis)
Source: BMC Plant Biol. 2019 Apr 25;19:154. doi: 10.1186/s12870-019-1744-8 (PMC6482567; doi:10.1186/s12870-019-1744-8)
Supplement: Supplementary file 5 — Table S4. Details of the Gene Ontology annotation. (DOCX 21 kb) [file 12870_2019_1744_MOESM5_ESM.docx]

| Table S4 Details of the Gene Ontology annotation | | |
| --- | --- | --- |
| Name | Gos | Annotations |
| PeTTF1 | GO:0003682 | Molecular_function:Chromatin binding |
| PeTTF3 | GO:0003677 | Molecular_function:DNA binding |
| PeTTF5 | GO:0008652 | biological_process:cellular amino acid biosynthetic process |
| PeTTF7 | GO:0003682;GO:0003677 | Molecular_function:Chromatin binding and DNA binding |
| PeTTF9 | GO:0003682 | Molecular_function:Chromatin binding |
| PeTTF12 | GO:0008652 | biological_process:cellular amino acid biosynthetic process |
| PeTTF14 | GO:0008652 | biological_process:cellular amino acid biosynthetic process |
| PeTTF21 | GO:0003682 | Molecular_function:Chromatin binding |
| PeTTF23 | GO:0003677 | Molecular_function:Chromatin binding and DNA binding |
| PeTTF24 | GO:0003682 | Molecular_function:Chromatin binding |
| PeTTF31 | GO:0003682 | Molecular_function:Chromatin binding |
