# Supplementary material for: The trihelix family of transcription factors: functional and evolutionary analysis in Moso bamboo (Phyllostachys edulis)
Source: BMC Plant Biol. 2019 Apr 25;19:154. doi: 10.1186/s12870-019-1744-8 (PMC6482567; doi:10.1186/s12870-019-1744-8)
Supplement: Supplementary file 6 — Table S6. Design the qRT-PCR primers. (DOCX 25 kb) [file 12870_2019_1744_MOESM6_ESM.docx]

Table S6 Design the qRT-PCR primers

| PeTTF1 | Forward primer | CGGCTGCCAGGAACGACT |
| --- | --- | --- |
|  | Reverse primer | CGCTCCGCCTCCCTCTTA |
| PeTTF2 | Forward primer | TCGGGGTAGGGTTCTTGAGG |
|  | Reverse primer | CGAGTCGAGTAGCGTCTGGGTA |
| PeTTF3 | Forward primer | GAGCGTAGGCTGGAGATGGC |
|  | Reverse primer | CGAGTCGAGTAGCGTCTGGGTA |
| PeTTF4 | Forward primer | TATTGCCTGTCTCCCTTATCCC |
|  | Reverse primer | ACTGCACGCCGAACTTGTG |
| PeTTF5 | Forward primer | TATTGCCTGTCTCCCTTATCCC |
|  | Reverse primer | ACTGCACGCCGAACTTGTG |
| PeTTF6 | Forward primer | TATTGCCTGTCTCCCTTATCCC |
|  | Reverse primer | ACTGCACGCCGAACTTGTG |
| PeTTF7 | Forward primer | GGGGTAGGGTTCTTGAGGATGG |
|  | Reverse primer | TGCTGCGAGTCGAGTAGCGT |
| PeTTF8 | Forward primer | GGGGTAGGGTTCTTGAGGATGG |
|  | Reverse primer | TGCTGCGAGTCGAGTAGCGT |
| PeTTF9 | Forward primer | GAGGGAGGTAATGTTGTGGACG |
|  | Reverse primer | TGCTGGAACTCGGAGACGC |
| PeTTF10 | Forward primer | GAGGGAGGTAATGTTGTGGACG |
|  | Reverse primer | TGCTGGAACTCGGAGACGC |
| PeTTF11 | Forward primer | CCCTTATCCCCTTTTGTTCTCA |
|  | Reverse primer | CGCTCGGCACGGTACTCTT |
| PeTTF12 | Forward primer | CGCTACAAGGTGGAGTGCCA |
|  | Reverse primer | CGGGGAGTTGCCGACGAT |
| PeTTF13 | Forward primer | GGCAAGACTAATCACCACCACC |
|  | Reverse primer | TTCATCCGCTGCCATCCA |
| PeTTF14 | Forward primer | GGCAAGACTAATCACCACCACC |
|  | Reverse primer | TTCATCCGCTGCCATCCA |
| PeTTF15 | Forward primer | CCCGTCTGCGGACCTCTT |
|  | Reverse primer | CGTCGTCGTCATAGTCGTTGTT |
| PeTTF16 | Forward primer | GGCAAAGCACGCCAGGAA |
|  | Reverse primer | GGGTGGTAGGGGTTGGAGAAG |
| PeTTF17 | Forward primer | CAAAGCACGCCAGGAAGCA |
|  | Reverse primer | GGGTGGTAGGGGTTGGAGAAG |
| PeTTF18 | Forward primer | CAAAGCACGCCAGGAAGCA |
|  | Reverse primer | GGGTGGTAGGGGTTGGAGAAG |
| PeTTF19 | Forward primer | CAAAGCACGCCAGGAAGCA |
|  | Reverse primer | GGGTGGTAGGGGTTGGAGAAG |
| PeTTF20 | Forward primer | CAAAGCACGCCAGGAAGCA |
|  | Reverse primer | GGGTGGTAGGGGTTGGAGAAG |
| PeTTF21 | Forward primer | CAAAGCACGCCAGGAAGCA |
|  | Reverse primer | GGGTGGTAGGGGTTGGAGAAG |
| PeTTF22 | Forward primer | CAAAGCACGCCAGGAAGCA |
|  | Reverse primer | GGGTGGTAGGGGTTGGAGAAG |
| PeTTF23 | Forward primer | CAAAGCACGCCAGGAAGCA |
|  | Reverse primer | GGGTGGTAGGGGTTGGAGAAG |
| PeTTF24 | Forward primer | AATCACCACCACCATCATCACA |
|  | Reverse primer | TCCAGAGCCCTTCTTTCCCT |
| PeTTF25 | Forward primer | AATCACCACCACCATCATCACA |
|  | Reverse primer | TCCAGAGCCCTTCTTTCCCT |
| PeTTF26 | Forward primer | GCGAAACAAAAGCAGGCACT |
|  | Reverse primer | GCGGCAATGGAGGAAACC |
| PeTTF27 | Forward primer | GCGAAACAAAAGCAGGCACT |
|  | Reverse primer | GCGGCAATGGAGGAAACC |
| PeTTF28 | Forward primer | GCGAAACAAAAGCAGGCACT |
|  | Reverse primer | GCGGCAATGGAGGAAACC |
| PeTTF29 | Forward primer | GCGAAACAAAAGCAGGCACT |
|  | Reverse primer | GCGGCAATGGAGGAAACC |
| PeTTF30 | Forward primer | GCGAAACAAAAGCAGGCACT |
|  | Reverse primer | GCGGCAATGGAGGAAACC |
| PeTTF31 | Forward primer | GCGAAACAAAAGCAGGCACT |
|  | Reverse primer | GCGGCAATGGAGGAAACC |
| PeTTF32 | Forward primer | GCGAAACAAAAGCAGGCACT |
|  | Reverse primer | GCGGCAATGGAGGAAACC |
| PeTTF33 | Forward primer | GCGAAACAAAAGCAGGCACT |
|  | Reverse primer | GCGGCAATGGAGGAAACC |
| PeTTF34 | Forward primer | GCGAAACAAAAGCAGGCACT |
|  | Reverse primer | GCGGCAATGGAGGAAACC |
| PeTTF35 | Forward primer | GCGAAACAAAAGCAGGCACT |
|  | Reverse primer | GCGGCAATGGAGGAAACC |
