# Supplementary material for: The trihelix family of transcription factors: functional and evolutionary analysis in Moso bamboo (Phyllostachys edulis)
Source: BMC Plant Biol. 2019 Apr 25;19:154. doi: 10.1186/s12870-019-1744-8 (PMC6482567; doi:10.1186/s12870-019-1744-8)
Supplement: Supplementary file 7 — Table S5. Subcellular localization of TTF gene in Moso bamboo. (DOCX 21 kb) [file 12870_2019_1744_MOESM7_ESM.docx]

Table S5 Subcellular localization of TTF gene in Moso bamboo

| Gene | localization |
| --- | --- |
| PeTTF1 | chlo |
| PeTTF2 | chlo |
| PeTTF3 | chlo |
| PeTTF4 | nucl |
| PeTTF5 | chlo |
| PeTTF6 | nucl |
| PeTTF7 | chlo |
| PeTTF8 | chlo |
| PeTTF9 | nucl |
| PeTTF10 | nucl |
| PeTTF11 | nucl |
| PeTTF12 | cyto |
| PeTTF13 | nucl |
| PeTTF14 | nucl |
| PeTTF15 | nucl |
| PeTTF16 | nucl |
| PeTTF17 | nucl |
| PeTTF18 | mito |
| PeTTF19 | mito |
| PeTTF20 | nucl |
| PeTTF21 | nucl |
| PeTTF22 | chlo |
| PeTTF23 | chlo |
| PeTTF24 | nucl |
| PeTTF25 | nucl |
| PeTTF26 | mito |
| PeTTF27 | nucl |
| PeTTF28 | chlo |
| PeTTF29 | nucl |
| PeTTF30 | nucl |
| PeTTF31 | nucl |
| PeTTF32 | chlo |
| PeTTF33 | nucl |
| PeTTF34 | chlo |
